# Aging and putative frailty biomarkers are altered by spaceflight

**DOI:** 10.1038/s41598-024-57948-5

**Published:** 2024-06-11

**Authors:** Andrea Camera, Marshall Tabetah, Veronica Castañeda, JangKeun Kim, Aman Singh Galsinh, Alissen Haro-Vinueza, Ivonne Salinas, Allen Seylani, Shehbeel Arif, Saswati Das, Marcelo A. Mori, Anthony Carano, Lorraine Christine de Oliveira, Masafumi Muratani, Richard Barker, Victoria Zaksas, Chirag Goel, Eleni Dimokidis, Deanne M. Taylor, Jisu Jeong, Eliah Overbey, Cem Meydan, D. Marshall Porterfield, Juan Esteban Díaz, Andrés Caicedo, Jonathan C. Schisler, Evagelia C. Laiakis, Christopher E. Mason, Man S. Kim, Fathi Karouia, Nathaniel J. Szewczyk, Afshin Beheshti

**Affiliations:** 1 Intitute of Basic Medical Sciences, University of Oslo, Oslo, Norway; 2 Department of Agricultural and Biological Engineering, Purdue University, West Lafayette, IN 47907 USA; 3 Faculty of Medicine, Universidad de los Andes, Santiago, Chile; 4 Department of Physiology and Biophysics, Weill Cornell Medicine, New York, NY USA; 5 School of Medicine, Medical Sciences and Nutrition, University of Aberdeen, Aberdeen, AB24 3FX UK; 6 Biología, Colegio de Ciencias Biológicas y Ambientales COCIBA, Universidad San Francisco de Quito USFQ, Quito, Ecuador; 7 Escuela de Medicina, Colegio de Ciencias de La Salud COCSA, Universidad San Francisco de Quito USFQ, Quito, Ecuador; 8 Riverside-School of Medicine, University of California, Riverside, CA USA; 9 Center for Data-Driven Discovery in Biomedicine, Children’s Hospital of Philadelphia, Philadelphia, PA USA; 10 Division of Neurosurgery, Children’s Hospital of Philadelphia, Philadelphia, PA USA; 11 Atal Bihari Vajpayee Institute of Medical Sciences, Dr. Ram Manohar Lohia Hospital, New Delhi, India; 12 Department of Biochemistry and Tissue Biology, Institute of Biology, Universidade Estadual de Campinas, Campinas, SP Brazil; 13 Obesity and Comorbidities Research Center (OCRC), Universidade Estadual de Campinas, Campinas, SP Brazil; 14 Ohio Musculoskeletal and Neurological Institute, Heritage College of Osteopathic Medicine, Ohio University, Athens, OH 45701 USA; 15 Blue Marble Space Institute of Science, Seattle, WA USA; 16 Federal University of São Paulo (UNIFESP), São Paulo, Brazil; 17 Transborder Medical Research Center, University of Tsukuba, Ibaraki, 305-8575 Japan; 18 Department of Genome Biology, Faculty of Medicine, University of Tsukuba, Ibaraki, 305-8575 Japan; 19 Department of Botany, University of Wisconsin-Madison, Madison, WI USA; 20 Center for Translational Data Science, University of Chicago, Chicago, IL 60637 USA; 21 Clever Research Lab, Springfield, IL 62704 USA; 22 Northwestern University Feinberg School of Medicine, Chicago, IL 60611 USA; 23 Amazon Web Services, Osaka, Kita Ward Japan; 24 Department of Biomedical and Health Informatics, The Children’s Hospital of Philadelphia, Philadelphia, PA 19041 USA; 25 Department of Pediatrics, Perelman School of Medicine, University of Pennsylvania, Philadelphia, PA 19104 USA; 26 Translational-Transdisciplinary Research Center, Clinical Research Institute, Kyung Hee University Hospital at Gangdong, School of Medicine, Kyung Hee University, Seoul, South Korea; 27 Data Science Institute, School of Business, Universidad San Francisco de Quito USFQ, Quito, Ecuador; 28 Instituto de Investigaciones en Biomedicina iBioMed, Universidad San Francisco de Quito USFQ, Quito, Ecuador; 29 Mito-Act Research Consortium, Quito, Ecuador; 30 Colegio de Ciencias de la Salud, Escuela de Medicina, Universidad San Francisco de Quito USFQ, Quito, Ecuador; 31 McAllister Heart Institute and Department of Pharmacology, The University of North Carolina at Chapel Hill, Chapel Hill, NC USA; 32 Department of Oncology, Department of Biochemistry and Molecular and Cellular Biology, Georgetown University, Washington, DC USA; 33 The WorldQuant Initiative for Quantitative Prediction, Weill Cornell Medicine, New York, NY USA; 34 Blue Marble Space Institute of Science, Exobiology Branch, NASA Ames Research Center, Moffett Field, CA USA; 35 Space Research Within Reach, San Francisco, CA USA; 36 Center for Space Medicine, Baylor College of Medicine, Houston, TX USA; 37 Stanley Center for Psychiatric Research, Broad Institute of MIT and Harvard, Cambridge, MA USA; 38 Blue Marble Space Institute of Science, Space Biosciences Division, NASA Ames Research Center, Moffett Field, CA USA

**Keywords:** Genetics, Medical research

## Abstract

Human space exploration poses inherent risks to astronauts’ health, leading to molecular changes that can significantly impact their well-being. These alterations encompass genomic instability, mitochondrial dysfunction, increased inflammation, homeostatic dysregulation, and various epigenomic changes. Remarkably, these changes bear similarities to those observed during the aging process on Earth. However, our understanding of the connection between these molecular shifts and disease development in space remains limited. Frailty syndrome, a clinical syndrome associated with biological aging, has not been comprehensively investigated during spaceflight. To bridge this knowledge gap, we leveraged murine data obtained from NASA’s GeneLab, along with astronaut data gathered from the JAXA and Inspiration4 missions. Our objective was to assess the presence of biological markers and pathways related to frailty, aging, and sarcopenia within the spaceflight context. Through our analysis, we identified notable changes in gene expression patterns that may be indicative of the development of a frailty-like condition during space missions. These findings suggest that the parallels between spaceflight and the aging process may extend to encompass frailty as well. Consequently, further investigations exploring the utility of a frailty index in monitoring astronaut health appear to be warranted.

## Introduction

**Figure 1:**
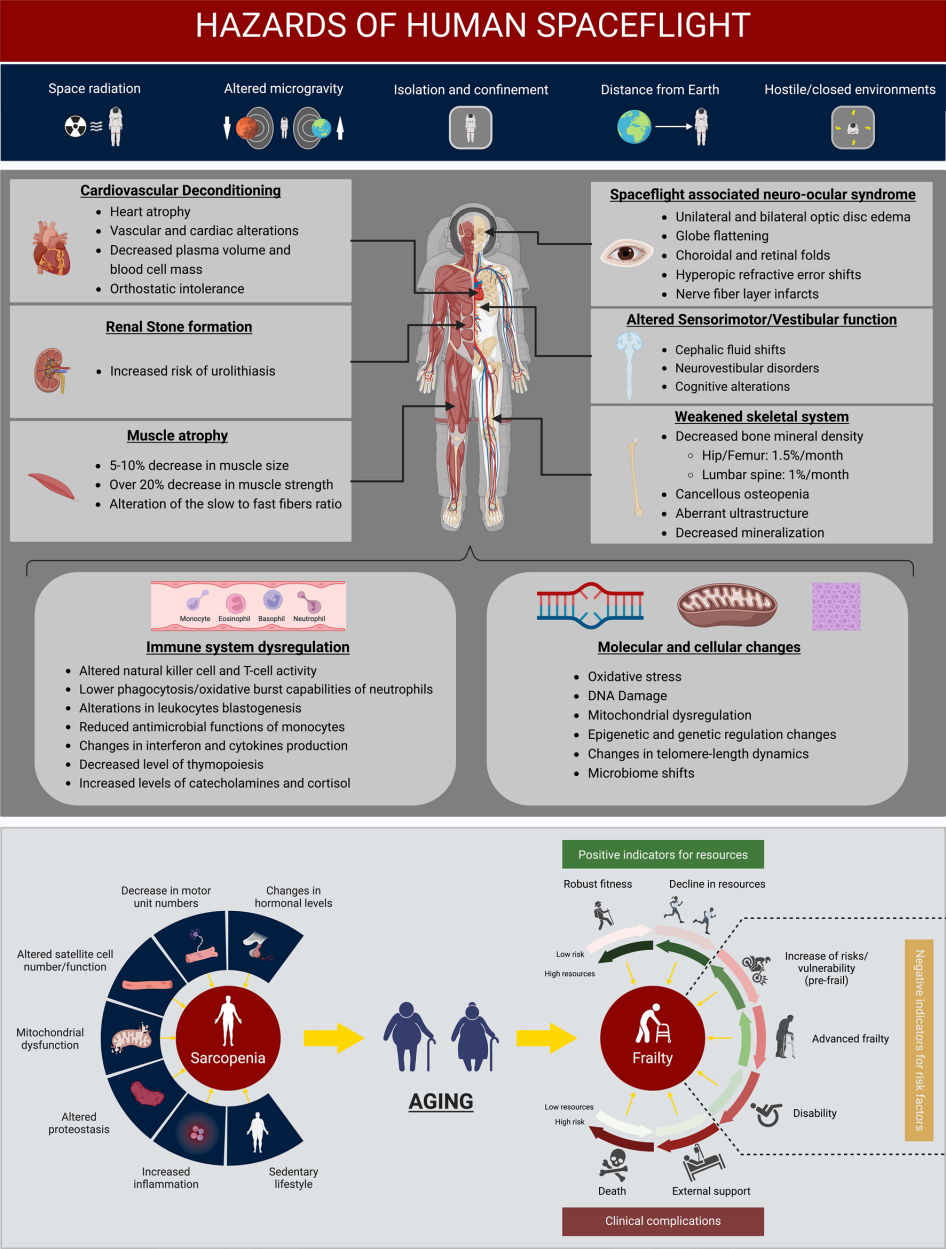
Effects of spaceflight and a model of aging. Human spaceflight presents five major challenges: space radiation, altered gravity, isolation and confinement, distance from Earth, and hostile/closed environments are hazards for crewed spaceflight. These stressors support pathophysiological alterations and cellular and molecular changes which could be involved in the development of biological aging and frailty in astronauts. Muscle loss in astronauts has a similar pathophysiology as sarcopenia in the elderly. Created using BioRender.

Missions beyond low Earth orbit are the new frontier of crewed space exploration. Future missions to Mars as well as long-duration missions to the Moon will be significantly more sustained than any previous deep space mission. During transit, the space environment presents key challenges for astronauts’ safety^1^. Previous literature describes microgravity, radiation exposure, isolation and confinement as major stressors that are able to induce pathophysiological changes in the heart, skeletal muscle, and immune system, as well as bone loss, central nervous system alterations, and increased cancer risk^2–4^. Thus, there is increasing interest to identify the molecular mechanisms driving those health risks, including changes in mitochondrial function, genetic and epigenetic regulation, telomere-length dynamics, DNA damage, and oxidative stress^3,5^ (Fig. 1). Interestingly, the molecular mechanisms of spaceflight-related stressors share similarities with the hallmarks of aging: mitochondrial dysfunction, genomic instability, epigenetic alterations, and telomere length changes (including brief elongation in flight and increased shorter telomeres post-flight), cellular senescence, and dysbiosis, among others^6^. As a defined set of aging-related molecular changes, the hallmarks of aging may be specifically studied to assess spaceflight impact on human physiology, which could induce a condition similar to premature or pathological aging^3^.

Aging is a state of depleted biological resilience resulting in an increased vulnerability to stressors. It leads to a systemic loss of the body’s capacity to maintain homeostasis and health^3^. The aging phenotype manifests in metabolic syndrome, cardiovascular disease, diabetes, neurological deterioration, and cancer^7^. Old age is also marked by immune dysregulation, which can result in a chronic low-grade inflammation state called *inflammaging*, a status associated with increased levels of pro-inflammatory markers in blood and tissues^8^. The deterioration in muscle quality and quantity is another feature of aging, characterized by altered myofiber metabolism and impaired satellite cell activity^9^. Loss of muscle mass increases the risk of developing other comorbidities, in a vicious cycle that leads to unhealthy aging. These traits are often associated with a multifactorial geriatric syndrome known as “frailty”^10^.

Frailty, a recent concept in aging science, can be defined as a syndrome caused by the combined effect of numerous age-related alterations. These alterations lead to a depletion of physiological reserve and/or deterioration of cognitive functions, resulting in an increased risk of morbidity and mortality. Frailty can be clinically diagnosed by the presence of three or more of the following components: unintentional weight loss over the last year, weakness, exhaustion, slow gait, and low physical activity level^11^. Other criteria for diagnosing and assessing frailty are functional factors such as grip strength and gait speed^12,13^. More holistic definitions of frailty also consider the presence of comorbidities, cognitive impairment, psychosocial risk factors and other common geriatric syndromes^14^. The use of clinical scores to quantify frailty has been attempted, but it is undermined by encountered limitations in terms of ethnicity, risk prediction, diagnosis, and prognosis. However, advancements in the understanding of the biological processes related to frailty and aging, along with the development of high-throughput techniques, allowed the development of novel assessment models based on “omics” biomarkers^15^.

Part of the deconditioning of human physiology caused by spaceflight resembles the features of frail patients, for instance the physical component related to muscle loss.

Sarcopenia is generally defined as a progressive age-related condition characterized by the loss of skeletal muscle quality, performance, strength, and mass^16^. When this condition is driven by aging, it is defined as primary sarcopenia^9^. Interestingly, muscle loss in astronauts shares similarities with sarcopenia in the elderly, especially during long duration spaceflight, and could be interpreted as a sarcopenia-like syndrome^17,18^.

The study of frailty traits in astronauts involves the investigation of aging’s biological pathways, which could be elicited by the space environment. Yet, despite the reported similarities between the features of frailty and consequence of spaceflight, it remains unclear whether the space environment can influence the onset of age-related dysfunctions in astronauts. Getting insights about the signatures of frailty and sarcopenia in spaceflight data is a key step towards the development of countermeasures to achieve safer crewed space missions and translate therapy to patients on Earth. Here, we hypothesize the emergence of aging, frailty and sarcopenia related transcriptomic signatures during and after spaceflight in astronauts, murine and cellular models. We investigate this occurrence using multi-omics and systems-informatic approaches. We analyzed transcriptomic data from rodent research missions flown to the International Space Station (ISS) (available from NASA’s Open Science Data Repository (OSDR), previously known as GeneLab^18^), astronaut data from a recent JAXA study, and data from the first civilian commercial spaceflight mission, Inspiration4 (i4). We identified altered expression in genes related to frailty and muscle loss, that may lead to an early frailty phenotype. Our results raise the possibility that exposure to the space environment leads to changes consistent with frailty, including inflammation, muscle wasting and other age-related features. Our findings propose a method to study the development of frailty-related health risks, which astronauts may develop during spaceflight, in the perspective of achieving adequate preventative measures.

## Results

### Multiple frailty related biomarkers are differentially expressed in rodent muscles during spaceflight

**Figure 2:**
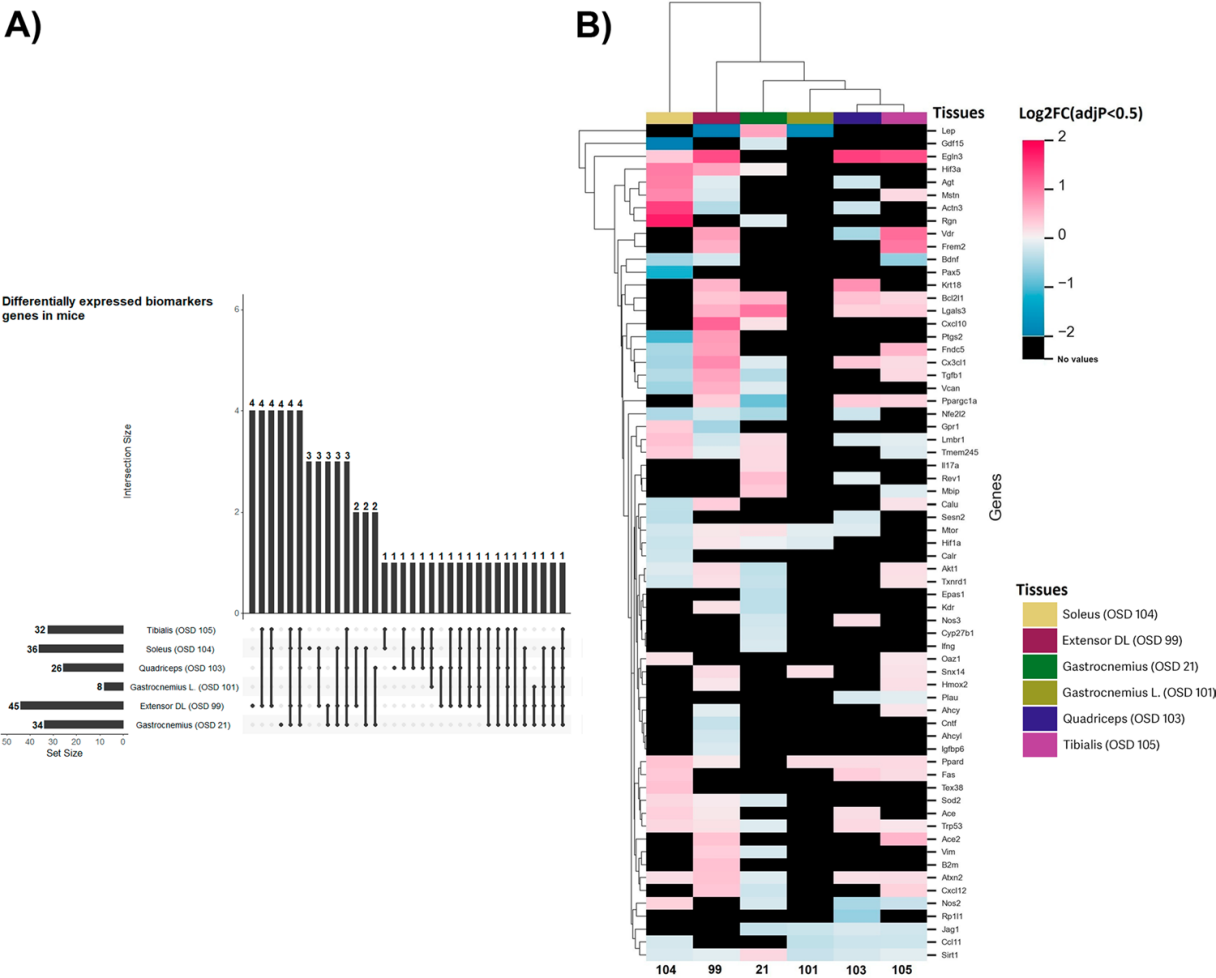
Frailty-related biomarkers are differentially expressed in rodent muscles during spaceflight. Putative frailty linked genes from NASA Open Science Data Repository (former GeneLab). The transcriptomic signature of spaceflight is investigated with differential expression analysis in multiple tissues. (**A**) Upset plots of overlapping differentially expressed frailty genes in rodent and human samples. (**B**) Heatmap of differential expression analysis for the frailty gene in human and rodent samples. Rodent samples comprise spaceflight skeletal muscle. Heatmap considers only DEG with adjusted *p-value* < 0.5. Black color indicates no value.

To determine the impact of frailty during spaceflight, we constructed, based on previous literature^19–22^, a list of putative frailty biomarker genes for humans and mice (Supplementary Data 1). Mouse (OSD-21, 99, 101, 103, 104, 105) datasets from OSDR were analyzed to identify differentially expressed genes (DEGs) in flight versus control condition with a statistical cut-off of adjusted *p*-value < 0.5. In mice, altered expression of frailty-related genes in the following tissues were identified: gastrocnemius (34 genes in OSD-21 and 8 genes in OSD-101); extensor digitorum longus (EDL) (45 genes in OSD-99); quadriceps (26 genes in OSD-101); soleus (36 genes in OSD-104); tibialis anterior (32 genes in OSD-105) (Fig. 2A). A maximum number of four frailty-related genes was also found to be unique to each tissue type and a maximum number of 4 was common between the different datasets (Supplementary Data 2). Hierarchical clustering of the overlapping gene expression across muscle types revealed a bias towards the up-regulation of frailty-related genes (Fig. 2B). As an example, the extensor digitorum longus had several upregulated genes (*EGLN3, PTGS2, VDR, FREM2, KRT18, BCL2L1, LGALS3, CXCL10, CX3CL1, FNDC5, TGFB1, CAN*, and *PPARGC1A*). Whereas the soleus (OSD-104) had relatively few downregulated genes (*GDF15, PTGS2, BDNF, PAX5, CX3CL1, FNDC5, VCAN, CALU*, and *SESN2*).

### Interferon inflammatory response and muscle tissue development pathways are enriched in rodent muscles during spaceflight

**Figure 3:**
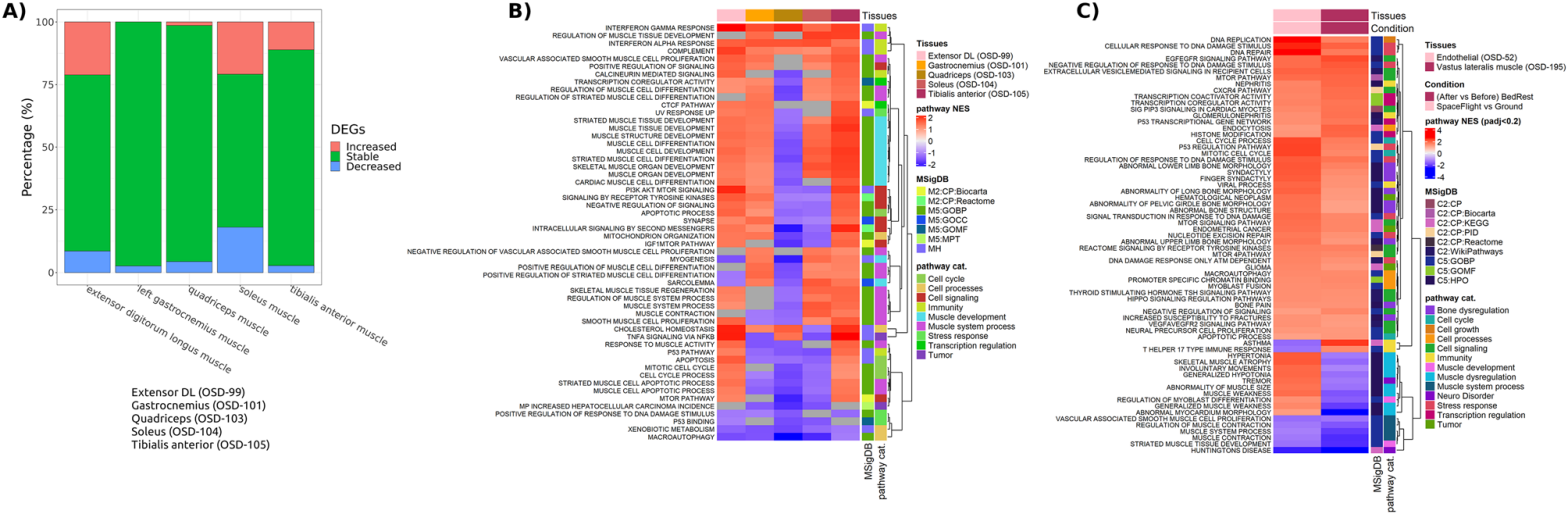
Inflammatory response pathways are enriched in rodent muscles during spaceflight. The transcriptomic signature of spaceflight is investigated with gene set enrichment analysis (GSEA) for putative aging-related pathways in multiple tissues. (**A**) Percentage of the differentially expressed genes which are stable, increased or decreased in rodent samples. (**B**) and (**C**) Heatmap of the normalized enrichment score for the enriched aging-related pathways in rodents and human samples. The dark gray locations in the heatmap indicate missing values for the NES, resulting from off-range adjusted *p*-values (padj) of the analysis. The assumed range is padj < 0.3.

To determine overall frailty impact of spaceflight on tissues Gene Set Enrichment Analysis (GSEA)^23^ analysis was performed on specific aging-related pathways (selected from the Molecular Signatures Database (MSigDB)^23^ (Supplementary Data 3). Rodent datasets showed a general enrichment of the pathways with an overall upregulation in EDL and tibialis anterior, downregulation in quadriceps, and a mixed regulation in gastrocnemius soleus (Fig. 3A,B). Summary themes of each functional cluster are displayed by the external color panel at the right side of sub-figure B and C. Despite a mixed direction of regulation, a clear enrichment of these pathways in the spaceflight group when compared to the control was evident across the datasets. The soleus muscle revealed an increase in the innate immune response inflammatory signature and concomitant downregulation of the IGF-1 pathway (Fig. 3B). Previous literature showed that the soleus muscle is the first to be impacted by spaceflight and also known to experience a significant dysregulation of mitochondrial and immune functions in space^24^. Immune response can downregulate IGF-1 anabolic activity, promoting muscle wasting^16^. Of note, this muscle shows the largest decline in mass in the RR1 mission and IGF-1 pathway might be involved^25^. Several putative aging-related pathways were enriched in human datasets (Fig. 3C), showing up-regulation in the majority of cases. Of note, interferon alpha and gamma response pathways are upregulated in all the datasets investigated. The increase in immune and inflammatory signatures we identified is consistent with various reports that associate chronic inflammation with frailty, although causality has yet to be established^8,26^. Nonetheless, our results could be useful for biomarkers related to spaceflight risk and consistent with clinical correlations of increased low-grade inflammation and muscle wasting^16^.

### Novel sarcopenia-related genes are differentially expressed during spaceflight

Sarcopenia is a condition associated with frailty. In our analysis, the best predictors of sarcopenia were genes that are part of autophagic and protein degradation processes. After studying databases from 118 people with and without sarcopenia (GSE111006, GSE111010, and GSE111016)^27^, 6,892 DEGs were identified by performing Mann–Whitney U tests^28^ on gene expression data for every single gene (i.e., 65,217 genes) in a pair-wise manner across samples from both sets of patients (Supplementary Data 4). A simple classifier (i.e., k-nearest neighbors) was then used to estimate individual predictive power for that condition^29^. Next, via co-expression network analysis upon these DEGs, the most highly correlated module (i.e., BROWN = 0.93) to sarcopenia was found. We used a pathway and gene ontology analysis upon BROWN to curate a list of 21 genes that were significantly enriched in biological processes related to sarcopenia^30^.

**Figure 4:**
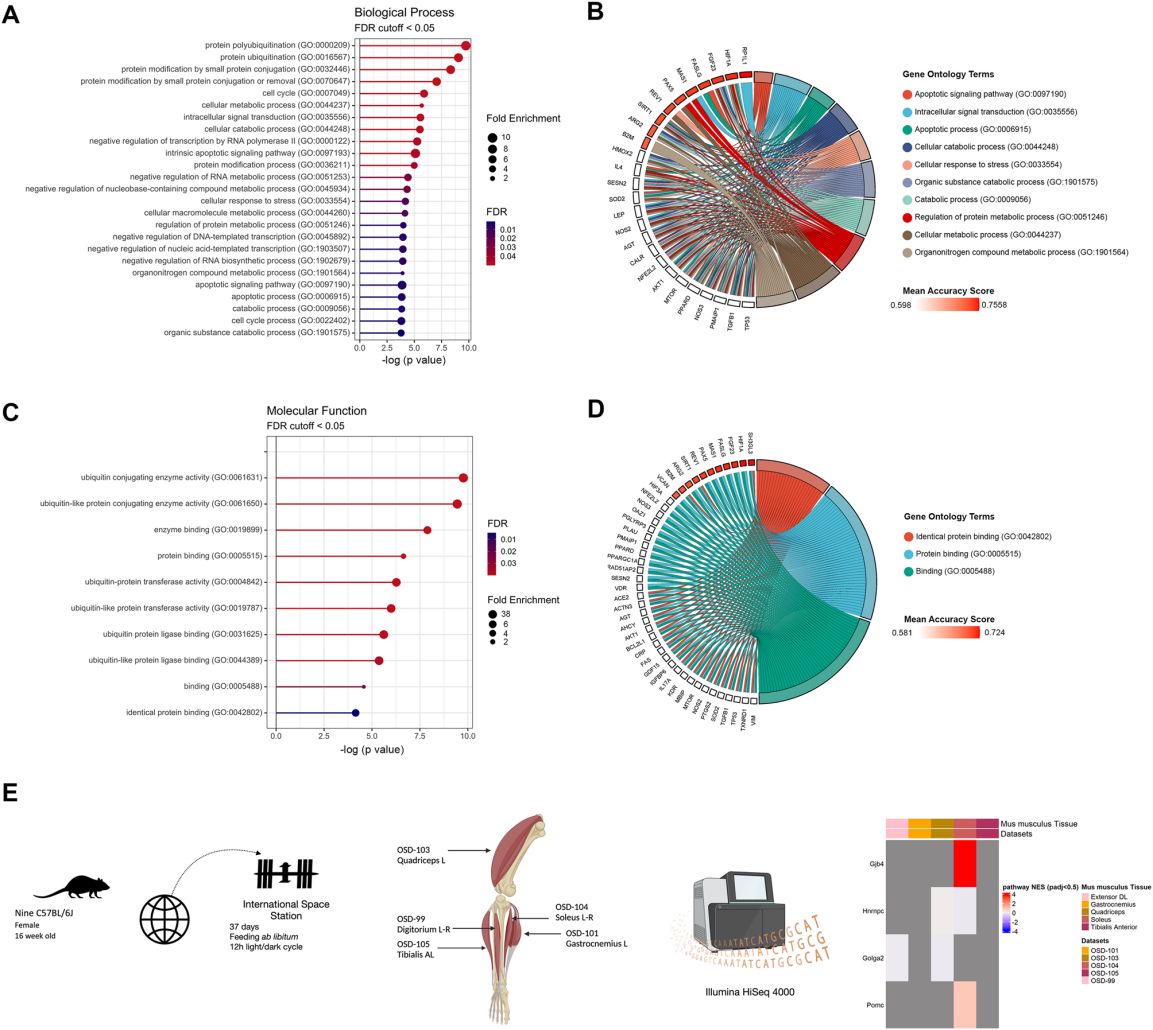
Evidence of shared catabolic pathways between sarcopenia and frailty markers and their differential expression in space-flown mice. (**A**) Significantly enriched Biological Processes using a curated biomarker gene list obtained by the overlap of three gene sets studying sarcopenia (superseries GSE111017: GSE111006, GSE111010, and GSE111016) defined through a Mann- Whitney analysis. (**B**) The frailty biomarkers found to be part of ten GO Biological Processes terms, from which R1PL1 had the highest Mean Accuracy Score (MAS) score. (**C**) Significantly enriched Molecular Functions using a curated biomarker gene list. (**D**) Similarly, three GO Molecular Function terms were found to be a shared pathway with the defined frailty biomarkers from which SH3GL3 had the highest MAS score. (**E**) Schematic of the data utilized for the heatmap showing the four genes out of the 21 sarcopenia frailty genes that were present in the murine data sets. Heatmap considers only DEG with p < 0.05.

Here, we found that the frailty biomarkers list was enriched in Biological Processes Gene Ontology (BP GO) terms in a very similar manner to those found with sarcopenic biomarkers alone (Fig. 4A)^29^. In addition to BP GO, the same was true for molecular functions (MF) GO term enrichment (Fig. 4C). Interestingly, we found that eight of the biomarkers identified for frailty had the ability to predict sarcopenia in GSE111006, GSE111010, and GSE111016 with a Mean Accuracy Score (MAS) of > 0.65 (*RP1L1, SH3GL3, HIF1A, FGF23, FASLG, MAS1, PAX5*, and *REV1*) (Fig. 4B,D).

Using the sarcopenia gene expression classifier we had established above, we re-examined the existing datasets for alterations in the 21 genes. To do so, we took the expression data from the murine datasets (EDL (ODS-99), left gastrocnemius (ODS-101), quadriceps (ODS-103), soleus (ODS-104), and tibialis anterior (ODS-105)) and evaluated the expression of our sarcopenia classifier (Fig. 4E). We found that only *GJB4, HNRNPCL1, GOLGA2* and *POMC* were DEGs in at least one of the datasets. *GJB4* is a connexin (Cx) gene encoding the gap junction protein CX30.3^31^. *HNRNPCL1* plays a role in consolidating the nucleosome and neutralizing core hnRNPs proteins^32^. *GOLGA2* encodes the GM130 protein necessary for the assembly of the Golgi apparatus. Interestingly, mutations in *GOLGA2* lead to neuromuscular disorders and muscular dystrophy^33^. *POMC* codes for the precursor protein proopiomelanocortin producing active peptides generating melanocyte stimulating hormones (MSHs), corticotropin (ACTH) and β-endorphin. POMC deficiency leads to adrenal failure and obesity^34^. Of note, the dataset from the soleus muscle in mice (OSD-104), demonstrated to have a significant overexpression of *GJB4, POMC* and significant downregulation of *HNRNPC* (*p* < 0.05).

### Multiple frailty related biomarkers are differentially expressed in human muscles during spaceflight

**Figure 5:**
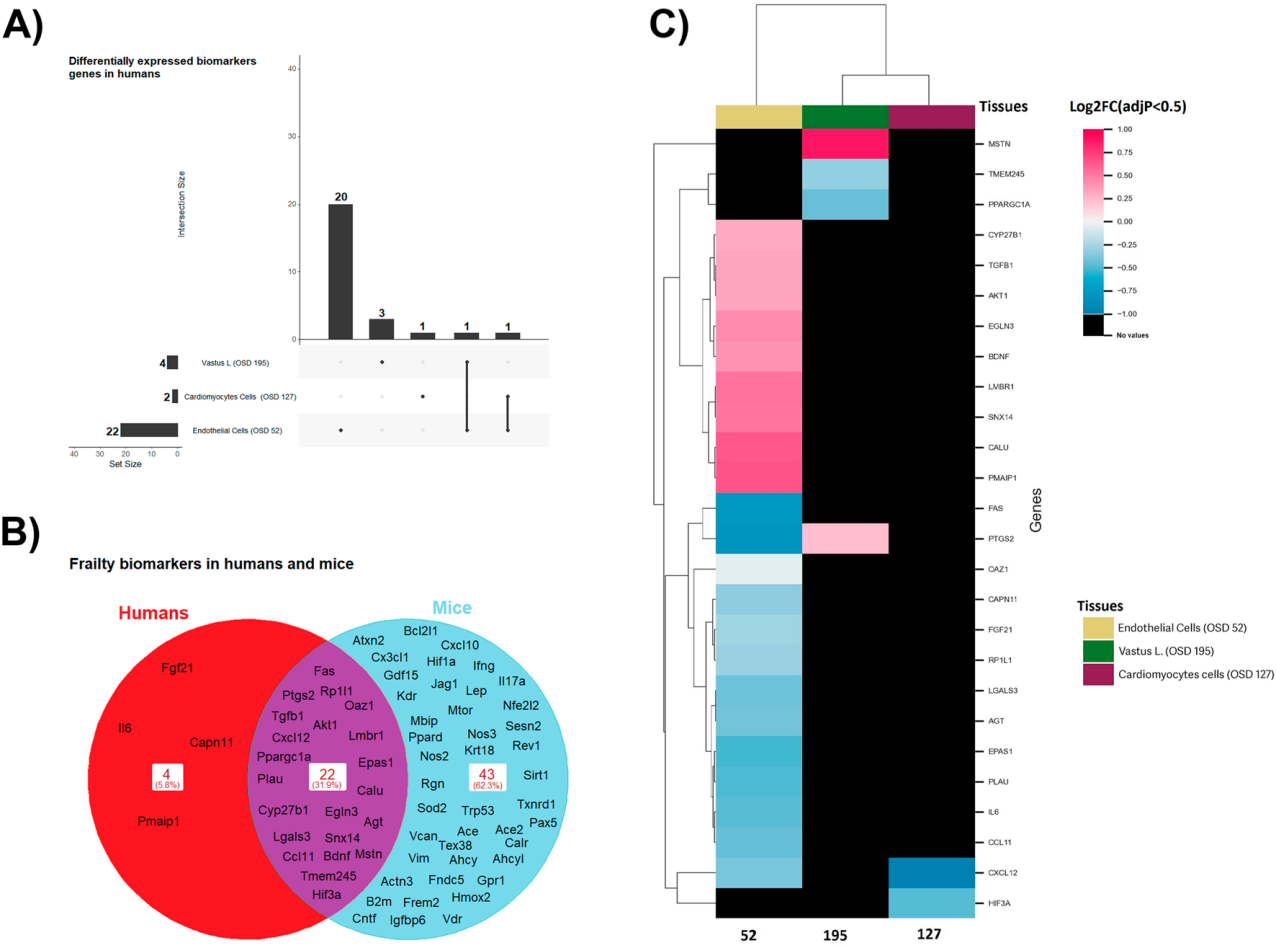
Frailty-related biomarkers are differentially expressed in humans during spaceflight and ground-based spaceflight simulated conditions. Putative frailty linked genes from NASA Open Science Data Repository (former GeneLab). The transcriptomic signature of spaceflight is investigated with differential expression analysis in multiple tissues. (**A**) Upset plot of overlapping differentially expressed frailty genes in human samples. (**B**) Venn diagram of differentially expressed frailty genes in rodent and human samples shows the common differentially expressed genes between the two species. (**C**) Heatmap of differential expression analysis for the frailty gene in human samples. Human samples comprise spaceflight human umbilical vein endothelial cells, bed rest skeletal muscle cells and cardiac progenitors differentiated from human pluripotent stem cells in 3D culture under simulated microgravity. Heatmap considers only DEG with adjusted *p-value* < 0.5. Black color indicates no value.

We applied the same list of putative frailty biomarker genes (Supplementary Data 1) to investigate differentially expressed genes in Open Science Dataset’s human samples as in Fig. 2. OSD-52 and 195 were analyzed to identify differentially expressed genes (DEGs) in flight, on random positioning machine or in bed rest versus control condition with a statistical cut-off of adjusted *p*-value < 0.5. Vastus lateralis muscle (OSD-52), cardiac progenitors (OSD-127) and endothelial cells (OSD-195) showed 22, 2 and 4 frailty-related genes, respectively (Fig. 5A).

We compared the differential expression profiles between mice and human dataset. Approximately a third of the frailty genes were conserved between humans and mice, which suggests that the murine models can provide good translation to human biology (Fig. 5B). Out of 73 differentially expressed frailty-related genes, 22 (32%) were common in humans and mice (Fig. 5B). Forty-three (62%) were unique to mice and 4 (6%) were unique to only humans. In humans, 9 frailty genes were upregulated and 13 were downregulated in the vastus lateralis muscle (Fig. 5C and Supplementary Data 2).

Several downregulated genes were associated with immunity-related pathways, while most upregulated genes were associated with metabolism and Vitamin K or D pathways. In endothelial cells, two genes were downregulated, and two were upregulated. The downregulated genes, *TMEM245* and *PPARGC1A*, are associated with the cell-membrane and gluconeogenesis, while the upregulated genes, *MSTN* and *PTGS2*, are associated with regulation of skeletal muscle growth and prostaglandin biosynthesis (Supplementary Data 5)^35^. While there is  no direct link between gluconeogenesis and frailty, both are related to the body's response to stress and maintaining homeostasis. Diabetes, a condition that affects glucose metabolism, has been linked to frailty^36,37^. In diabetes, the body's ability to regulate blood glucose levels is impaired, potentially impacting gluconeogenesis. Frail individuals, who have a diminished ability to resist stressors, may be more susceptible to the effects of these metabolic imbalances^38^.

### Multiple frailty related biomarkers are differentially expressed in astronauts

**Figure 6:**
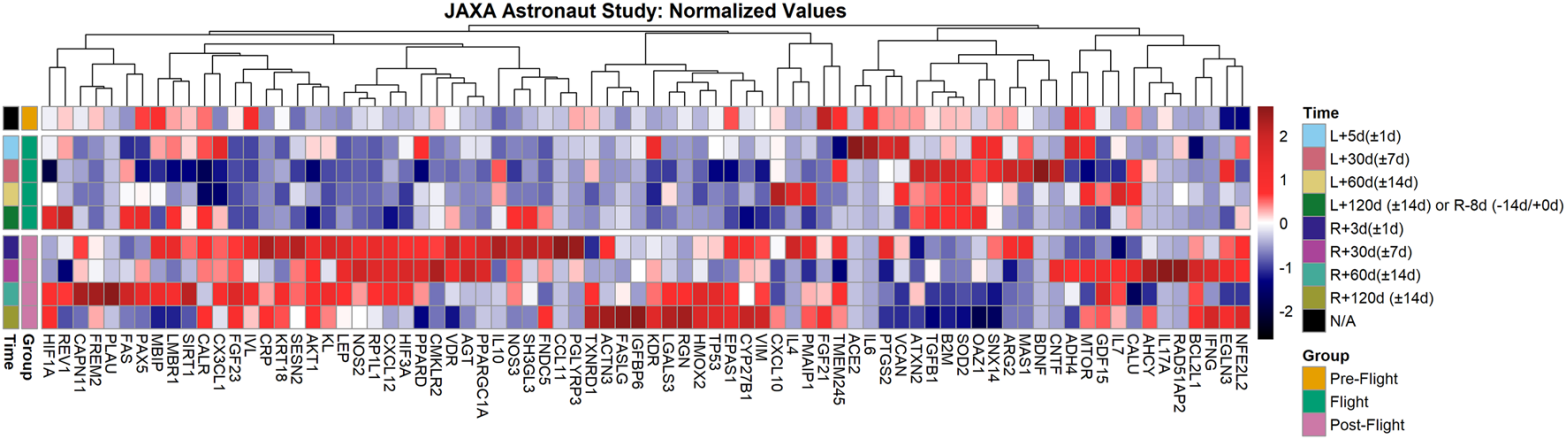
Frailty-related biomarkers are differentially expressed in astronauts exposed to 120-days of Low Earth Orbit Spaceflight. Putative frailty linked genes from JAXA Cell-Free Epigenome (CFE). Heatmap of the normalized plasma cell-free RNA expression values for the frailty genes over time for the six astronauts over 120 days in space from JAXA study. The values shown are the averaged normalized expression values for all six astronauts for each time point during flight and post-flight. The three pre-flight time points were averaged together, since the changes for genes in the time leading up to flight are considered to be the same and part of the baseline values. For the time, L = Launch (i.e., meaning time after launch from Earth and the number indicates length in space) and R = Return to Earth.

Having confirmed altered aging and frailty signatures in largely rodent transcriptomic data, we wanted to test if frailty biomarkers were also altered in astronauts. To enable this analysis, we used two recent studies^39^. First, using astronaut data from JAXA plasma cell-free RNA profiling study, we examined the changes occurring in RNAs from the frailty biomarker genes between pre-flight, in-flight, and post-flight (i.e., after return to Earth) (Fig. 6). Our RNA analysis reveals a global response of frailty-related gene expression to the space environment, which is characterized by in-flight and post-flight expression changes. Most of the genes investigated were subject to changes when compared to pre-flight conditions, either during spaceflight or later after return to Earth. A large number of genes that were reduced during spaceflight showed an increase after re-entry (e.g., *AKT1, NOS2, FGF23*, and *HIF3A*). Conversely, several genes show an opposite behavior and tended to be reduced during spaceflight, and underwent reduction after re-entry (e.g., *TGFB1, B2M, NOS1, AOC1, SOD2, SOD3*, and *OAZ1*).

Interestingly, cell-free RNAs from several genes (e.g,. *FGF23, KRT18, AKT1, B2M, NOS1, AOC1, SOD2 and SOD3*) did not return to the pre-flight baseline levels, even after 120 days. The data suggest that space conditions alter the HIF1 pathway which stimulates the various molecular or cellular processes related to hypoxia-responsive genes such as *HIF1A, HIF1AN, ARNT, ARNT2, NOS1, NOS2, NOTCH1* and *RBX1*, that are known to regulate a wide variety of cellular physiology including metabolic reprogramming, anti-apoptosis, migration, proliferation, amyloid β production and prion stabilization^40,41^. An interesting observation emerging from the data is the increased cell-free RNA signature of *HIF1A* and *HIF3A* post-flight. Hypoxia Inducible Factor (HIF) is a key regulator of immune cell function^42^, and its dysregulation could alter immune response. We also observe an increase of RNAs derived from several nitric oxide (NO) related genes, which are biologic mediators in multiple processes, such as in neurotransmission and microbial and antitumoral activities. It is understood that nitric oxide (NO) is a key vasodilator in the cardiovascular system and its synthesis is catalyzed by the enzyme family nitric oxide synthases (NOS), neuronal (NOS1), inducible (NOS2) and endothelial synthases (NOS3)^43^. *NOS1* and *NOS2* are constitutively expressed by tubules of the human kidney, while *NOS3* is expressed by endothelial cells and is implicated with the formation and maintenance of vascularized tissues. Furthermore, *AKT1* plays a role in the signal transduction of growth factors, as well as in cell survival, cellular senescence, and aging. There is evidence that AKT signaling is associated with an imbalance of phosphatidylinositide 3-kinases that is altering the aged brain^44^. Chronic AKT activation intensified aging-induced cardiac hypertrophy in murine heart tissues^45^. In connection to phosphate intake, FGF23 is known to be secreted from the skeletal system and influence the kidneys through the klotho gene receptor36. The upregulation of HIF-related genes could be interpreted through findings from earlier studies which have implicated the HIF pathway with the impairment of energy-dependent cellular processes, and mutations in mitochondrial DNA which accelerate aging processes^41,46^.

**Figure 7:**
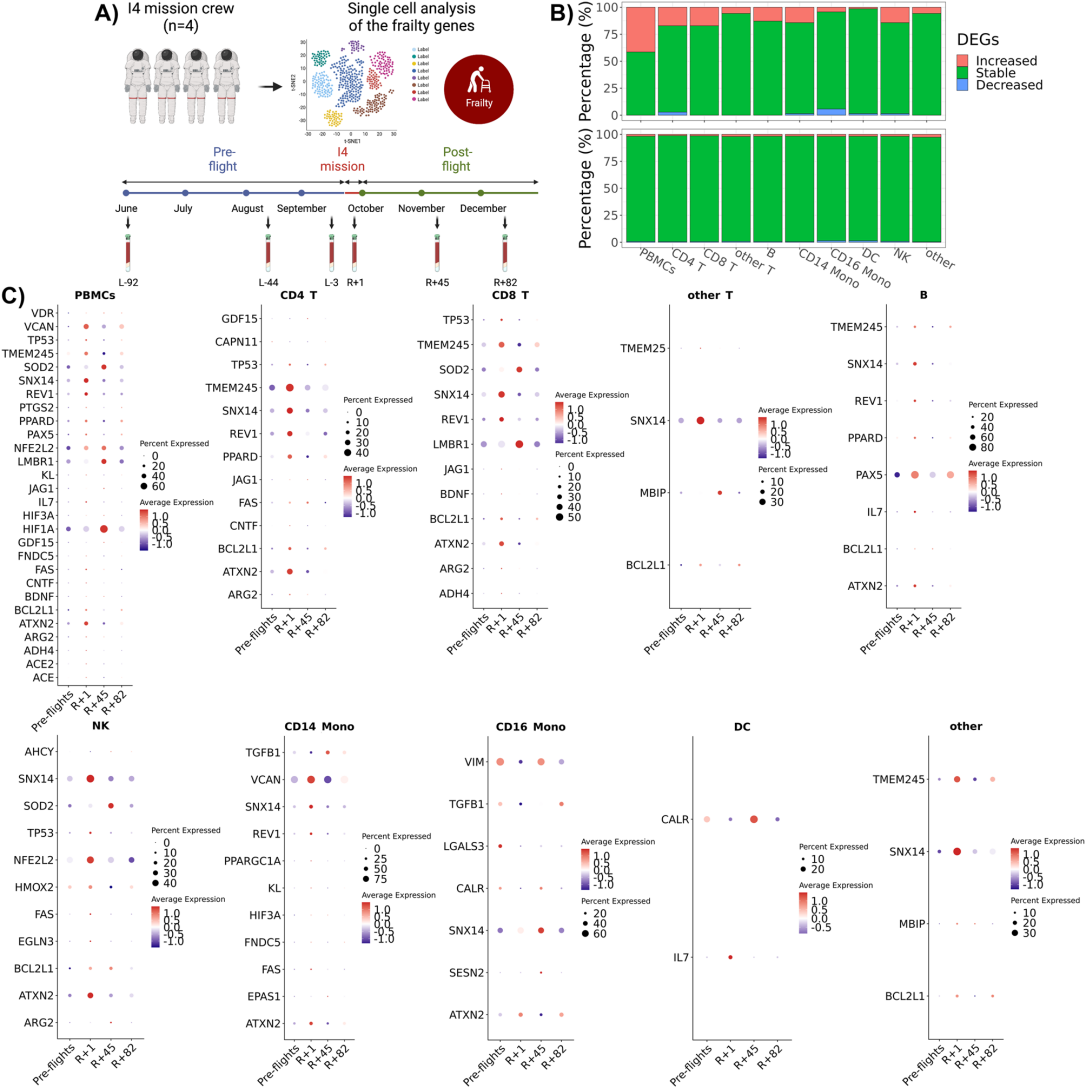
Frailty-related biomarkers are differentially expressed in astronauts exposed to 3-days of Low Earth Orbit Spaceflight. Frailty linked genes from Inspiration4 (i4) human peripheral blood mononuclear cells (PBMCs). (**A**) Schematic of the i4 experiments and the samples utilized for this analysis. (**B**) The overall percentage of up (i.e., increased), down (i.e., decreased), and no change (i.e., stable) expressed frailty genes in the i4 data (top plot) compared the overall gene distribution (bottom plot). (**C**) Dot plot of the single cell RNA expression for the frailty genes over time for the 4 astronauts over 3 days in space from the i4 civilian crew mission. The image shows the differential expression values for each cell type in analysis. The values are based on expression for each time point before-flight and post-flight. However, data from samples collected just after reentry (R + 1) is considered spaceflight condition. For the time, L = Launch, R = Return to Earth, the number + n is the time (in days) after L or R.

Next, we used data from the first civilian commercial 3-day space mission (referred to as Inspiration4 (I4), to examine the impact of short-duration spaceflight on putative frailty biomarker transcriptomic signature^47^. From the I4 mission, single-cell gene expression data from peripheral blood mononuclear cells (PBMCs) were generated and compared across multiple timepoints (Fig. 7A). Frailty genes were increased in PBMCs and subpopulations post-flight compared to pre-flight timepoints, and the percentage of the increased genes were higher than the percentage of differentially expressed genes (DEGs) (Fig. 7B). The percentage of increased frailty genes was the highest in PBMCs, lowest in dendritic cells (DCs), and similar in the remaining subpopulations (Fig. 7B). Generally, the average expression and percentage of expression of the increased genes were increased at R + 1 compared to pre-flights (L-92, L-44, L-3) and returned to baseline over time (Fig. 7C). For example, several genes were upregulated in various pathways at R + 1 compared to pre-flight and reverted to baseline over time. Implicated pathways include: immunity (*ARG2, PPARD*), EGFR trafficking (*ATXN2*), regulators of apoptosis (*BCL2L1, FAS*), survival factor for neuronal cell types (*CNTF*), cell–cell signaling (*JAG1*), metabolism (*PPARD*), DNA repair *(REV1)*, neuronal excitability and synaptic transmission (*SNX14*), structural component of sarcomeric Z-line *(TMEM245)* and cell cycle regulation *(TP53)* (Fig. 7C).

### Metabolic flux simulations of gene expression changes reveal dysregulation of metabolic functions related to frailty

**Figure 8:**
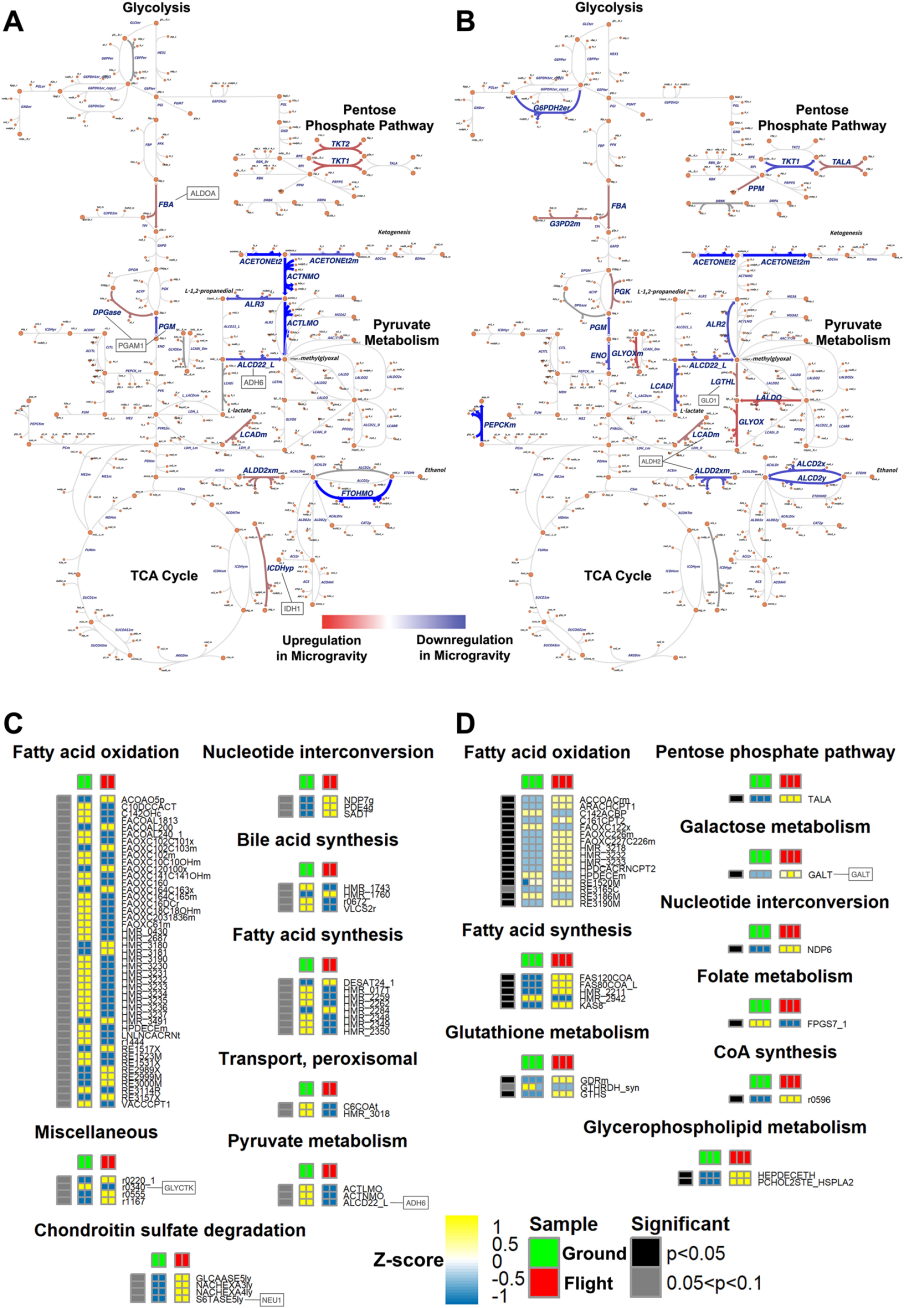
Metabolic flux simulation analysis on OSD-91 and OSD-127. (**A**) and (**B**) Overview of carbohydrate metabolism illustrated by custom-made Escher [81] for OSD-91 and OSD-127, respectively. The associated pathways (i.e., TCA Cycle, Glycolysis, Pentose phosphate pathway, Pyruvate metabolism) whose metabolic reactions with relative activations are demonstrated. The red color presents the upregulated metabolic fluxes in flight and the blue color represents the downregulated fluxes. (**C**) and (**D**) Heatmaps showing relative metabolic flux rates (rows) versus human samples (columns) for OSD-91 and OSD-127, respectively. Only particular pathways demonstrating significant alteration of metabolic flux rates are listed, where the blue to yellow heatmap color scales indicate row-wise Z-scores for those flux rates. The leftmost bar represents differential testing results between Flight and Ground in *p* values < 0.05 (black) or p values between 0.05 and 0.1 (gray) through the Van Der Waerden test. Genes in the boxes are enzymes showing significantly different expressions for their corresponding reactions.

Having found alterations in gene expression associated with aging and frailty and knowing that biologic systems are dynamic, we used a subset of the gene expression to examine dynamic changes in metabolism. We applied our updated, context-specific, metabolic models that performed custom-made flux balance analysis (FBA) simulations. Here, we used two different transcriptional changes (RNA-seq) between flight and ground (OSD-91 (GSE65943) for cultured human TK6 lymphoblastoid cells; and OSD-127 (E-GEOD-84582) for cardiomyocytes from human pluripotent stem cells) (Fig. 8).

In TK6 lymphoblastoid cells, microgravity led to transcriptional changes through altered methylation patterns. These transcriptional changes, in turn, altered the oxidative stress and carbohydrate metabolism pathways^48^. However, the flux simulation analysis showed that other pathways associated with lipid metabolism, fatty acid oxidation, fatty acid synthesis, and bile acid synthesis, are downregulated during flight. While chondroitin sulfate degradation, nucleotide interconversion, and peroxisomal transport are upregulated. Considering the carbohydrate metabolism aspect of the flux simulation analysis, only pyruvate metabolism (end product of glycolysis) showed significantly altered expression in microgravity (Figs. 8A,C).

By contrast, the other metabolic flux simulation displayed marked up-regulation during flight in lipid metabolism associated pathways: fatty acid oxidation, fatty acid synthesis, and glycerophospholipid metabolism (Figs. 8B,D). The cells also exhibited increased galactose metabolism, nucleotide interconversion, Coenzyme A (CoA) synthesis, glutathione metabolism, as well as pentose phosphate pathway in carbohydrate metabolism. The only significant downregulation in microgravity was detected in folate metabolism. This cardiomyocyte study (using 3D tissue engineering of cardiac progenitors from human pluripotent stem cells) found increased gene expression levels associated with growth, development, and survival for cardiac progenitors in microgravity^49^.

## Discussion

To our knowledge, no prior links to frailty-like signatures have been observed in the context of spaceflight, and these markers represent an important direction for future research, especially as mission durations increase to years. In recent years, human space research has benefitted from collecting and integrating diverse types of biological data, such as genomics, proteomics, and metabolomics, and storing them in biobanks for future analysis^19,50^. These multi-omics data and biobanks continue to provide valuable insights into the aging-like processes in astronauts during long-term space missions and help identify potential biomarkers and interventions to counteract them^25,51–53^. In our manuscript, we include data from NASA’s OSDR, the SpaceX Inspiration4 and the JAXA astronaut study. These initiatives are hallmarks in the development of data banking strategies such as the Space Omics and Medical Atlas (SOMA)^54^. The NASA Twins study along with the JAXA Cell-Free Epigenome and Inspiration4 data reported here and elsewhere (Figs. 6 and 7) highlight the emerging ability to conduct omic analyses on astronaut samples ^27,39,55^. We propose the use of omics data to investigate the development of aging-like phenomena in spaceflight, which can also help improve our understanding of aging metrics like clonal hematopoiesis^56^, and also link to a wide array of new data from the SOMA package of data and papers ^48,53,55,55,57–65^.

Space medicine research has been mostly focused on the acute and medium-term effects of spaceflight on human physiology. However, some attributes related to the exposure to the space environment resemble aging-like pathophysiology: DNA damage, oxidative stress, and mitochondrial dysfunction^3^. Because of these similarities, observations from the aging field can be useful to understand the biological changes driven by spaceflight. In contrast with aging on Earth, it is unclear whether those molecular modifications last after reentry, and for this reason we refer to them as aging-like phenomena. Nevertheless, despite acute events such as space motion sickness and autonomic imbalance resolving after astronaut’s return to ground, the effects of these aging-like alterations might be clinically manifest in a chronic exposure scenario, like a long-duration mission to Mars, and could potentially trigger chronic disease development.

Studies in genetic/genomic aging models such as *C. elegans* and clinical evidence have demonstrated that the control of lifespan and health span are not identical^49,53^; therefore, there is an increasing need to identify markers able to determine the biological age of an individual. One proposed marker is the frailty index, which can serve as a better health indicator than chronological age, translating what is suggested by previous biological and clinical literature^7^. Here, we examined if aging and putative frailty biomarkers change during spaceflight. Our results from both rodent and human samples suggest that alterations indeed occurred in aging-related pathways to a different extent depending on the tissue. Importantly, we also found changes in expression of putative frailty genes, raising the possibility that these could be used as indicators of physiological changes during spaceflight much as they are being examined for utility in clinical assessment of physiological changes with age on Earth. Beyond health assessment, the ability to conduct -omic studies on astronauts allows for combining astronaut and model organism datasets in order to conduct truly translational research in space. Such studies would expand our understanding of molecular control of (patho)physiological changes in response to spaceflight.

The expression of putative frailty related biomarkers in JAXA Cell-Free Epigenome samples is altered during spaceflight, compared to ground control, as shown in Fig. 6; interestingly, several gene expression levels do not return to baseline after reentry. Although their expression could revert to normal after a longer period, this altered expression could hint to the presence of spaceflight-induced modifications that persist for longer than expected. For example, *AKT1* is upregulated at all timepoints after reentry. A persisted upregulation of the gene is linked to age related cardiac disease and dementia in animal models^45,46^. The presence of biomarker gene expression that does not revert to normal challenges the concept that every physiological change induced by spaceflight is reverted after astronauts’ return to Earth. This notion is not novel, for example, a recent report showed the development of distinct mutational profiles in astronauts^66^; on these bases, it would be worth monitoring these biomarkers for longer timepoints after reentry, and to exclude the possibility that they underlie any sub-clinical disease development. Permanent modifications after exposure to important stressors happen in patients as well. For instance, admission to Intensive Care Unit and severe COVID-19 have been associated with epigenetic changes that might alter aging-related gene expression^45,46^.

In addition to long-term spaceflight exposure, short-duration spaceflight data such as those provided by the i4 mission are also relevant to detect early signs of frailty development. In fact, frailty is not only associated to long term chronic disease pathways: clinical research hints that short term exposure to stressors such as COVID-19 hyperinflammatory response are capable of developing frailty-associated traits^56^. Regardless, longer spaceflight duration might be required to detect wider alterations in putative frailty biomarkers and clinically manifested phenotypes in astronauts; this highlights the need for further studies and validation.

Differential expression of frailty genes in astronauts’ cell-free DNA and PBMCs (Figs. 6 and 7) is not inherently the same as what is observed in the Open Science Dataset’s (OSDs) human samples (Fig. 5). Despite frailty signals potentially being detected in astronaut’s blood, it is not feasible to directly pair this result with the differential expression of frailty-related genes in muscle biopsies, due to the absence of matched data.

As previously suggested, this represents an opportunity to apply cutting-edge clinical research tools for monitoring and researching astronaut health. Indeed, genome sequencing onboard the ISS is currently used in the microbiological monitoring program^67^, paving the way for use in astronaut studies on the ISS. Previous studies of astronaut health have largely been conducted using standard clinical markers such as blood panels. While there is an emerging blood panel for assessment of frailty^68^, these would need to be processed on Earth. By contrast, the assessment of a future clinically validated transcriptomic panel for frailty could be conducted on ISS or other spacecrafts. Regardless of which panel or where such a panel is assessed, our findings suggest that future studies aimed at establishing such a validated model for astronauts could be fruitful areas of investigation for monitoring and improving astronaut health in- and post-flight. The establishment and validation of such a panel could either be done by correlating with physiological measures already captured by NASA’s standard measures program or correlating with physiological measures adapted from the emerging frailty indexes.

One of the most pronounced findings in both rodent and human datasets is the induction of interferon alpha and gamma responsive pathways. Interferons are cytokines associated with modulation of the immune response which is a known consequence of spaceflight^69^. Our observation of the activation of interferon pathways (Fig. 3) suggests that the activation of the innate immune system is a conserved mammalian response to spaceflight and aging. In the aging field, the concept of inflammaging suggests that the chronic low-grade dysregulation of the immune system that happens with aging underpins the development of diseases such as atherosclerosis, type II diabetes, and sarcopenia^7^. Indeed, at least ten clinical biomarkers of inflammation correlate well with the frailty index^70^.

Making the inflammaging parallel to spaceflight, it could be that an increased low-grade inflammatory response during spaceflight drives and/or worsens frailty and/or related processes (for example muscle wasting)^67,71^. Low grade inflammation contributes to the development of endothelial dysfunction and atherosclerosis on Earth^7,72^. In addition, radiation exposure can also lead to the development of cardiovascular disease in case of occupational exposure, as well as in cancer patients undergoing radiation therapy^69,71,73^. In astronauts, atherosclerosis could be promoted by an increased inflammatory status promoted by different stressors of the space environment (Fig. 1), and radiation exposure would play a primary role for it. Moon and Mars missions have a different radiation environment compared with the ISS, as astronauts traveling beyond Low Earth Orbit are potentially exposed to higher doses of galactic cosmic rays and solar particle event radiation^69^. Elevated radiation exposure promotes multiple dysregulations, causing cellular senescence in the endothelium, mitochondrial dysfunction and inflammation, driving endothelial dysfunction^71^. Exposure to higher doses of space radiation beyond low Earth orbit could also have a different, and perhaps increased, impact on frailty-related hallmarks and genes. Concerning endothelial dysfunction, an interplay between nitric oxide metabolism, production of reactive oxygen species and radiation exposure could play a role in the development of radiation-induced cardiovascular disease, as well as other disease pathways, in space^74,75^. Moreover, a pro-inflammatory and oxidative environment might facilitate the development of the spaceflight associated neuro-ocular syndrome (SANS)^76^; this condition would also increase the disability status of the crew.

Across species, altered metabolism is one of the most consistently reported responses to spaceflight. Unsurprisingly, these changes vary from organism to organism among microbes and tissue to tissue in animals (Fig. 8). A recent rodent transcriptomic study confirmed that the typical mammalian coordination of metabolism between the liver and muscles on Earth continues to function similarly in flight^77^. This metabolic axis is a key determinator of health with age, whose disruption is associated with obesity, diabetes, and frailty. Emerging techniques such as metabolomics are beginning to address the role and mechanisms of metabolic alteration in human muscle with age^73^. Here we have employed another emerging analytical approach, metabolic flux modeling from transcriptomic data (Fig. 8). Our results confirm that metabolic flux analysis successfully demonstrated metabolic alterations as expected, dependent on tissue type. Importantly, while our results confirm past observations that mitochondrial and metabolic changes are conserved features of space flown tissues and organisms^3^, they also demonstrate that the functional changes via metabolic flux are more important than the simple global up versus down changes often employed with reductionist approaches. The effect of microgravity exposure may cause specific mitochondrial protein adaptations that affect skeletal muscle tissue, namely 3-hydroxyacyl-CoA dehydrogenase activity, palmitate oxidation and cytochrome c oxidase enzymatic activity^75,78,79^. It is noteworthy that tissues might respond differently to microgravity and the mitochondrial bioenergetics adaptations seen in skeletal muscle may be different from the ones seen in cardiac muscle; however new sequencing data may provide an insight into these differences^18,75^. Thus, using data from multiple tissues and time points begins to provide a more comprehensive understanding of the mechanisms underlying the development of frailty in response to spaceflight. Importantly, our results demonstrate that these approaches can be employed directly in astronauts and/or in combination with model systems.

Muscle decline is another conserved feature between spaceflight and aging. It is also thought to contribute to frailty. Recently, functional measures of muscle decline with age have been well correlated with evolutionarily conserved gene expression changes with age^27,74^. In humans, changes in metabolic gene expression appear to occur prior to changes in inflammatory gene expression, suggesting that aging human muscle undergoes metabolic remodeling prior to becoming inflamed and sarcopenic—also highlighting the importance of temporal/dynamic data^71^. Similarly, in humans subjected to bedrest, an analogue for spaceflight, changes in metabolic gene expression and metabolic activity are an early event tied to altered intramuscular calcium levels^80^. The recent study of muscle-liver metabolic cross talk in mice used the same mouse muscle datasets we used for this manuscript (Fig. 8) and found altered glucose and lipid metabolism in response to spaceflight, consistent with past literature on space flown mammalian muscle^77^. Combining this past analysis with our new analysis of sarcopenic gene expression changes, it is noteworthy that few sarcopenic gene expression changes are noted in the space flown muscles with more observed changes in the soleus, which was the muscle displaying the largest decline in mass^25^. Together, these results raise the possibility that metabolic changes may indeed precede the loss of muscle mass in spaceflight, as observed with age and bedrest on Earth. It remains an open question if, like sarcopenia, metabolic alterations occur earlier than the inflammatory alterations in flight and whether inflammation or an altered metabolism is the key driver for a frailty-like phenotype in space. Lastly, given the similarities between transcriptomic changes with age and spaceflight, the facts that estrogen receptor has recently been proposed to be a key regulator of muscle gene expression changes with age and that estrogen receptor pathways have recently been shown to display differences in flight, raise the possibility that estrogen receptor pathways are key modulators of muscle health in space^27,77,81^. The restriction of available sources poses a frequent challenge in the field of space medical and biological research. In order to adequately tackle our research inquiry with comprehensive information, we adopted a diverse range of data from various public sources. It is essential to note that each individual experimental model incorporated does not entirely encapsulate the complete biology of astronauts exposed to space conditions. Therefore, caution is advised when extending conclusions from one model system to another or from cellular and tissue-level observations to the overall phenotypes of organisms. Nonetheless, a noteworthy aspect of our findings is the utilization of a weight-of-evidence approach, wherein data across multiple models are analyzed. This method reveals conserved pathways that consistently emerge in independent datasets, likely indicating fundamental biological responses akin to frailty in the context of spaceflight. Pursuing the development of biobanks for multi-omics data will deeply benefit space medicine and biology research. Yet, concurrently, bridging the gap between molecular changes, pathophysiological processes and phenotype development requires multi-omics biological data to be associated with individual physical metrics. This is crucial to investigate complex syndromes such as frailty. Future studies will be conducted to observe global and systemic impact on how the frailty index will change during spaceflight.

## Methods

### Ethical statement

All human studies were done with ethical approvals with established and approved IRBs at Weill Cornell Medicine. Blood samples were provided by SpaceX Inspiration4 crew members after consent for research use. The procedure followed guidelines set by Health Insurance Portability and Accountability Act (HIPAA) and operated under Institutional Review Board (IRB) approved protocols and informed consent was obtained. Experiments were conducted in accordance with local regulations and with the approval of the IRB at the Weill Cornell Medicine (IRB #21-05023569).

### Analyses of NASA open science data repository datasets: differential gene expression and GSEA pathway analysis

All murine data were obtained from NASA Open Science Data Repository (former GeneLab), where they were previously collected and shared by other investigators. No live animals were used in this investigation. The Open Science Data (OSD) we analyzed are: OSD-21, OSD-52, OSD-99, OSD-101, OSD-103, OSD-104, OSD-105 and OSD-195. Information regarding the chosen datasets is provided in Table 1.

**Table 1:** Genelab datasets analyzed in the manuscript.

Identifier	Title	Authors, title, publisher, version, DOI	Analyzed tissue
OSD-21	Effects of spaceflight on murine skeletal muscle gene expression	Barth J. "Effects of spaceflight on murine skeletal muscle gene expression", NASA Open Science Data Repository, Version 3, http://doi.org/10.25966/c36b-3g68	Gastrocnemius muscle
OSD-52	Expression data from SPHINX (SPaceflight of Huvec: an INtegrated eXperiment)	Bradamante S, Versari S, Longinotti G, Barenghi L, Maier J. "Expression data from SPHINX (SPaceflight of Huvec: an INtegrated eXperiment)", NASA Open Science Data Repository, Version 4, http://doi.org/10.26030/nt3p-p547	Endothelial
OSD-99	Rodent Research-1 (RR1) NASA Validation Flight: Mouse extensor digitorum longus muscle transcriptomic and epigenomic data	Galazka J, Globus R "Rodent Research 1", GeneLab, Version 4, http://doi.org/10.26030/1h3m-3q49	Extensor digitorum longus muscle
OSD-101	Rodent Research-1 (RR1) NASA Validation Flight: Mouse left gastrocnemius muscle transcriptomic, proteomic, and epigenomic data	Galazka J, Globus R "Rodent Research 1", GeneLab, Version 5, http://doi.org/10.26030/sdmt-ae51	Gastrocnemius muscle
OSD-103	Rodent Research-1 (RR1) NASA Validation Flight: Mouse quadriceps muscle transcriptomic, proteomic, and epigenomic data	Galazka J, Globus R "Rodent Research 1", GeneLab, Version 4, http://doi.org/10.26030/9vzk-b116	Quadriceps muscle
OSD-104	Rodent Research-1 (RR1) NASA Validation Flight: Mouse soleus muscle transcriptomic and epigenomic data	Galazka J, Globus R "Rodent Research 1", GeneLab, Version 4, http://doi.org/10.26030/em9r-w619	Soleus muscle
OSD-105	Rodent Research-1 (RR1) NASA Validation Flight: Mouse tibialis anterior muscle transcriptomic, proteomic, and epigenomic data	Galazka J, Globus R "Rodent Research 1", GeneLab, Version 4, http://doi.org/10.26030/xgw6-6t64	Tibialis anterior muscle
OSD-195	Effects of 21 days of bedrest on human skeletal muscle gene expression	Rullman E. "Effects of 21 days of bedrest on human skeletal muscle gene expression", NASA Open Science Data Repository, Version 1, http://doi.org/10.26030/r6bv-rk07	Vastus lateralis muscle
OSD-202	Low dose (0.04 Gy) irradiation (LDR) and hindlimb unloading (HLU) microgravity in mice: brain transcriptomic and epigenomic data	Mao X. "Low dose (0.04 Gy) irradiation (LDR) and hindlimb unloading (HLU) microgravity in mice: brain transcriptomic and epigenomic data", NASA Open Science Data Repository, Version 8, http://doi.org/10.26030/ewfb-7g23	Brain

The Genelab datasets are re-analyzed to produce the t-score. The Differential Expression (DE) analysis of RNA-Seq datasets (OSD-99, OSD-101, OSD-103, OSD-104, OSD-105) is performed using DESeq2 version 1.26.0^82^, in R software version 4.1.2. Expected counts from the RSEM step were extracted and rounded up to the next integer and used as input for DE analysis. The Differential Expression (DE) analysis of microarray datasets (OSD-21, OSD-52, OSD-195) is performed using the package limma, in R version 4.1.2^74^. The Ensembl IDs of the genes in the DE datasets were annotated to their corresponding gene symbols using the R package biomaRt^83,84^.

We performed gene set enrichment analysis (GSEA) on the differentially expressed datasets using fGSEA package to determine to what extent aging and putative frailty pathways were impacted in spaceflight^85^. Tentative aging and frailty gene pathways were downloaded from the Molecular Signatures Database (MSigDB)—a joint project of UC San Diego and Broad Institute^23,86^. MSigDB is a multicollection of databases among which are databases of curated gene sets, and databases of ontology gene sets that are used in pathway analysis. The latest version MSigDB v2022.1 was used in the pathway search.

In search of pathways that are potentially related to frailty, in MSigDB, we entered keywords of processes that are common denominators to aging in different organisms^66^. The processes of interest are: changes in genomic and genetic material; cellular processes; nutrient signaling; and responses at the tissue level with bone and muscle as the tissues of interest. Changes in genomic and genetic material that are deemed to potentially associate with frailty include genome instability, epigenetic alterations, histone modifications, DNA methylation, DNA damage, and changes in chromatin structure. Cellular processes that are of interest to frailty pathways include: (1) processes that are key to maintaining the cell cycle—cell senescence, stem-cell generation, and the pathways that involve p53, the protein tumor suppressor; (2) mechanisms involved in proteostasis (protein regulation); (3) mitochondrial processes involving dysfunction and disease; and (4) intercellular processes that involve inflammation and intercellular communication. The actions of the protein complexes—proteasome, lysosome, and chaperone—are considered in the network of pathways that regulate proteins. Putative pathways of nutrient sensing and signaling include pathways mediated by insulin signaling and mTor signaling, and the role of Ampk.

The downloaded pathways were identified as putative frailty pathways when at least two (2) putative frailty biomarker genes were present among the genes that are involved in the pathway. This is applied from the following reasoning: if multiple genes that can possibly interact to express a phenotype are present in a pathway, then that pathway can be associated with that phenotype.

The outcome of the fGSEA analysis is the normalized enrichment score (NES) of any given pathway, alongside valuable information on the analysis, such as the adjusted p-value, and the leading-edge genes of the pathway. The NES is the enrichment score normalized based on the number of genes in the gene set. It indicates the representation of the pathway genes in the dataset gene list, which is priorly ranked according to the values of the t-score of the genes. A positive NES indicates that the pathway genes are mostly represented at the top of the gene list, while a negative NES indicates that the pathway genes are mostly represented at the bottom of the gene list. The plots of the NES of the different pathways for a given dataset are produced using ggplot2, and the heatmaps across different datasets or different genes are obtained using a complex heatmap R package^87,88^.

### Analysis on frailty biomarker genes and pathways

A list of Frailty Biomarkers was generated from a survey of literature that was produced from studies that were inspired by the FRAILOMICS Initiative. The biomarker genes for mouse (*Mus musculus*) were obtained as a literal translation from human biomarker genes using biomaRt.

Historically, frailty was assessed based on measuring functional phenotypic parameters. The Fried phenotype regards frailty as a biological syndrome that comprises five components, i.e., shrinking, weakness, fatigue, slowness, and a low physical activity level^20^; frailty is diagnosed when poor performance in three or more components are present. The Rockwood frailty index identifies frailty based on the accumulation of age-related deficits, including various symptoms, signs, functional impairments, and laboratory abnormalities; it is calculated as the ratio of the number of accumulated deficits divided by the total number of deficits measured^20^. The FRAILOMICS Initiative was introduced with the aim of developing validated measures comprising both classic and omics-based laboratory biomarkers.

Since the inception of initiatives such as the FRAILOMICS^70,89^, a number of approaches have been utilized to identify molecular biomarkers of frailty, among which are: the identification of differentially expressed biomarkers based on the risk of frailty^89^; the identification of genes regulated in “hallmarks of aging” pathways^19^; and the identification of molecular biomarkers that closely associate inflammatory mediators with frailty^35^.

In the publication by Durinck et al.^89^, subjects are stratified as frail or non-frail based on Fried’s definition, and frailty biomarkers are identified as those that are upregulated in frail subjects.

In the publication by Berrios et al.^19^, gene expression databases were searched to identify genes regulated in “hallmark of aging” pathways, namely (1) inflammation, (2) mitochondria and apoptosis, (3) calcium homeostasis, (4) fibrosis, (5) neuromuscular junction (NMJ) and neurons, (6) cytoskeleton and hormones. A total of 44 biomarkers were evaluated based on differential expression. The markers were categorized by priority scores, with a higher priority score reflecting an upregulation of the biomarker in several pathways.

In the publication by Lebrasseur et al.^22^, a meta-analysis was conducted with the aim of showing whether inflammatory mediators are overproduced in frail older adults. Forty-nine studies, published between 2002 and 2018 analyzing frailty and inflammation parameters, were selected for the meta-analysis. Among them, 35 studies (70%) associated frailty to CRP, 33 studies (66%) evaluated IL6, 13 studies (26%) reported data for TNFα, and 12 (24%) analyzed other different inflammation biomarkers, including IL10, soluble TNF receptors I (sTNF-RI) and II (sTNF-RII), intercellular adhesion molecule 1 (ICAM-1), monocyte chemoattractant protein-1 (MCP-1), and IL6 receptor (IL6-R). Regarding frailty identification, 92% of the studies employed Fried’s frailty criteria, 6% of the studies used the frailty index developed by Rockwood and Mitnitski, and the remaining small percentage of studies employed tools and indicators recently described in the literature.

In the work described in this paper, the authors have used the biomarker genes identified in the named studies as putative biomarker genes of frailty to conduct their analyses.

The differential expression (log2foldchange and padj < 0.5) of frailty biomarker genes were determined from the following two human datasets (OSD-52 and 195) and six mice datasets (OSD-21, 99, 101, 103, 104, 105). Subsequently, to generate the Venn diagram, R language v4.1.0^85^ was used, through the library ggplot2 v3.4.0^87^. It was extracted from the table of frailty genes (Table 2), with genes unique to mice and humans, and with genes common for both (the intersections). After generating the diagram, a list of genes was extracted and manually added to the diagram. To generate the two upset plot graphs (for mice genes and human genes), the R language^85^ was also used, using the library ggplot2 v3.4.0^83^ and ComplexUpset v1.3.5^90^. Finally, to generate the heatmap, the python 3 v3.10.0 language was used, through the Seaborn v0.12.1 and Clustermap v0.11.12 libraries. Two heatmaps were generated (one for the mice genes and the other for the human genes), relating the genes to the respective OSD datasets. To complement the heatmap analyses, a dendrogram has also been added for each graph.

**Table 2:** List of the frailty genes.

Mouse gene name	Mouse gene stable ID	Gene name	Human Gene stable ID
Ace	ENSMUSG00000020681	ACE	ENSG00000159640
Ace2	ENSMUSG00000015405	ACE2	ENSG00000130234
Actn3	ENSMUSG00000006457	ACTN3	ENSG00000248746
Adh4	ENSMUSG00000037797	ADH4	ENSG00000198099
Agt	ENSMUSG00000031980	AGT	ENSG00000135744
Ahcyl	ENSMUSG00000048087	AHCY	ENSG00000101444
Akt1	ENSMUSG00000001729	AKT1	ENSG00000142208
Arg2	ENSMUSG00000021125	ARG2	ENSG00000081181
Atxn2	ENSMUSG00000042605	ATXN2	ENSG00000204842
B2m	ENSMUSG00000060802	B2M	ENSG00000166710
Bcl2l1	ENSMUSG00000007659	BCL2L1	ENSG00000171552
Bdnf	ENSMUSG00000048482	BDNF	ENSG00000176697
Calr	ENSMUSG00000003814	CALR	ENSG00000179218
Calu	ENSMUSG00000029767	CALU	ENSG00000128595
Capn11	ENSMUSG00000058626	CAPN11	ENSG00000137225
Ccl11	ENSMUSG00000020676	CCL11	ENSG00000172156
Gpr1	ENSMUSG00000046856	CMKLR2	ENSG00000183671
Cntf	ENSMUSG00000079415	CNTF	ENSG00000242689
Crp	ENSMUSG00000037942	CRP	ENSG00000132693
Cx3cl1	ENSMUSG00000031778	CX3CL1	ENSG00000006210
Cxcl10	ENSMUSG00000034855	CXCL10	ENSG00000169245
Cxcl12	ENSMUSG00000061353	CXCL12	ENSG00000107562
Cyp27b1	ENSMUSG00000006724	CYP27B1	ENSG00000111012
Egln3	ENSMUSG00000035105	EGLN3	ENSG00000129521
Epas1	ENSMUSG00000024140	EPAS1	ENSG00000116016
Fas	ENSMUSG00000024778	FAS	ENSG00000026103
Fasl	ENSMUSG00000000817	FASLG	ENSG00000117560
Fgf21	ENSMUSG00000030827	FGF21	ENSG00000105550
Fgf23	ENSMUSG00000000182	FGF23	ENSG00000118972
Fndc5	ENSMUSG00000001334	FNDC5	ENSG00000160097
Frem2	ENSMUSG00000037016	FREM2	ENSG00000150893
Gdf15	ENSMUSG00000038508	GDF15	ENSG00000130513
Hif1a	ENSMUSG00000021109	HIF1A	ENSG00000100644
Hif3a	ENSMUSG00000004328	HIF3A	ENSG00000124440
Hmox2	ENSMUSG00000004070	HMOX2	ENSG00000103415
Ifng	ENSMUSG00000055170	IFNG	ENSG00000111537
Igfbp6	ENSMUSG00000023046	IGFBP6	ENSG00000167779
Il10	ENSMUSG00000016529	IL10	ENSG00000136634
Il17a	ENSMUSG00000025929	IL17A	ENSG00000112115
Il4	ENSMUSG00000000869	IL4	ENSG00000113520
Il6	ENSMUSG00000025746	IL6	ENSG00000136244
Il7	ENSMUSG00000040329	IL7	ENSG00000104432

### Inspiration4 (i4) sample collection

Inspiration4 was the world’s first all-civilian mission to orbit Earth. Four civilians, two males and two females, spent three days in low-Earth orbit (LEO) at 585 km above Earth. The mission launched from NASA Kennedy Space Center on September 15th, 2021, and splashed down in the Atlantic Ocean near Cape Canaveral on September 18th, 2021. Several human related experiments were carried out to study the effects of spaceflight on human health and performance in collaboration with SpaceX, the Translational Research Institute for Space Health (TRISH) at Baylor College of Medicine (BCM), and Weill Cornell Medicine. The experiments conducted on the Inspiration4 crew members were performed in accordance with the relevant guidelines and experimental protocols were approved at the principal investigators’ institutions at Weill Cornell Medicine. All I4 crew members have given informed consent to participate in these studies. The i4 crews’ physiological changes were monitored with ultrasound scanning, molecular diagnostics devices, smartwatch wearables devices, and etc.^91^. Infection or inflammation are the most prevalent clinical symptoms found during the long-term (around 6 months) spaceflight^92^. However, there were no clinical reports of infection or inflammation. Although two astronauts presented with space motion sickness, most metrics (e.g., internal jugular vein size, heart rate, complete blood count, gene expression, and cytokines) were either stable, or quickly reverted back to baseline after landing. The experiments conducted by the Inspiration4 crew members were performed in accordance with the relevant guidelines at the principal investigators’ institutions. Moreover, the different study designs and the corresponding methods to collect and analyze the biological samples were approved by the corresponding institutional IRB. All biological data derived from the Inspiration4 mission were collected pre and post flights. For this study, only data from blood samples were used. Pre-flight samples were collected at L-92, L-44, and L-3 days prior to launch to space. Upon return, post-flight samples were collected at R + 1, R + 45, and R + 82 days.

### i4 PBMC Single cell sequencing and analysis

Blood samples were collected before (Pre-launch: L-92, L-44, and L-3) and after (Return; R + 1, R + 45, and R + 82) the spaceflight. Chromium Next GEM Single Cell 5’ v2, 10 × Genomics was used to generate single cell data from isolated PBMCs. Subpopulations were annotated based on Azimuth human PBMC reference^93^. Dot plots are generated by the Seurat R package.

### JAXA cell-free epigenome (CFE) study RNA quantification data

Aggregated RNA differential expression data and study protocols were shared through NASA’s Open Science Data Repository with accession number: OSD-530^39^. Plasma cell-free RNA samples for RNA-seq analysis were derived from blood samples collected from 6 astronauts before, during, and after spaceflight on the ISS. Mean expression values were obtained from normalized read counts of 6 astronauts for each time point. Heatmaps were made for the frailty genes on the normalized values per time point using R package pheatmap version 1.0.12.

### Quantification of genes associated with sarcopenia

The curated short gene list of predictors of sarcopenia was obtained by analyzing a population of age matched individuals with and without sarcopenia using the expression superserie GSE111017. This list contains 21 genes that can predict sarcopenia in patients with an accuracy > 75%. The method for obtaining the gene list was performed as previously described^29^. Here, we used GOstat 2022-11-03 on R version 4.2 to find enriched Biological Process and Molecular Function terms (Fig A and C). Using the obtained GO terms we found that some of the Frailty linked genes from JAXA CFE were also part of some of the same processes (Fig B and D). The figure was created using the GOplot package 1.0.2 and modified to display the MAS for each gene. Finally, to determine the relevance of the shared 21 genes between all gene lists (Fig E), we assessed their expression in the mice datasets OSD-99, OSD-101, OSD-103, OSD-104, and OSD-105. Finally, the heatmap was generated as before using python 3 v3.10.0 language, through the Seaborn v0.12.1 and Clustermap v0.11.12 libraries and a dendrogram has also been added for each graph.

### Metabolic flux simulation analysis

We ran metabolic flux simulation on all available metabolic reactions for each RNA sequencing sample while applying our custom-made context-specific constraint-based metabolic modeling approach significantly updated from^23,94,95^. This updated simulation model was constructed based on the RECON3D metabolic model^57^ whose metabolic reactions were subsetted through CORDA^96^ and Cobrapy^97^ with gene-reaction-rule, where enzyme expression levels are linked to their corresponding metabolic reactions. For CORDA, flux confidence parameters ranging from 3 to 1 were decided as; (i) 55%, 25%, and 20% for OSD-91; (ii) 45%, 40%, and 15% for OSD-127, in order to minimize standard deviation of flux-levels across samples from each group for all groups. Also, several essential pathways such as ‘Glycolysis/Gluconeogenesis’, ‘CoA Synthesis’, ‘CoA Catabolism’, ‘NAD Metabolism’, ‘Fatty Acid Synthesis’, ‘Fatty Acid Oxidation’, and ‘Biomass and Maintenance Functions’ were manually activated for the simulation model stability. Note that ‘Oxidative Phosphorylation’ and ‘Citric Acid Cycle’ were not included in the essential pathways to take into account mitochondrial dysregulation. Our metabolic model initially optimized for NAD biosynthesis whose capacity through all pathways of human was computed by estimating optimized ‘NAD sink’ reaction flux level where the sink was defined as large pools of metabolites importing metabolites from/to the system. Subsequently, optimization for all available reactions was conducted iteratively to compute flux levels of their corresponding reactions by Gurobi solver as LP whose reference manual can be found in https://www.gurobi.com^98^. While gene expression levels were applied into the model by each sample, the other parameters were maintained identically for all simulation, where an environment i.e., Jupyter notebook 6.1.5 on Python 3.7.7 was utilized. The simulation reported outcomes as flux levels of all available reactions through the custom-made flux balance analysis (FBA) analyses and the levels were analyzed as grouped variables for comparison between ‘Flight’ and ‘Ground’ groups. Since it is hard to presume variance or normality between ‘Flight’ and ‘Ground’, a non-parametric Van der Waerden (VdW) test was applied to properly compare their flux levels using the R matrixTests package (v. 0.1.9). The comparison is illustrated as heatmaps with row-wise Z-scores on flux levels per each reaction in Fig. 8. Tabular data of metabolic flux analysis is shown in supplement 6.

### Supplementary Information

Dataset S1.

Dataset S2.

Dataset S3.

Dataset S4.

Dataset S5.

Dataset S6.

Supplementary Legends.

## Data Availability

The RNA-seq data for the RR1 missions and the JAXA CFE data are available via the NASA Open Science Data Repository’s (OSDR)’s Biological Data Management Environment (https://osdr.nasa.gov/bio/repo) with accession IDs: OSD-21, OSD-99, OSD-101, OSD-103, OSD-104, OSD-105 and OSD-530. For the Inspiration4 data, the data have been uploaded to two data repositories: the NASA Open Science Data Repository (osdr.nasa.gov; comprised of NASA GeneLab and the NASA Ames Life Sciences Data Archive [ALSDA]), and the TrialX database. Identifiers for publicly downloadable datasets in the OSDR are documented below. Data can be visualized online through the SOMA Browser (https://epigenetics.weill.cornell.edu/apps/I4_Multiome/), the single-cell browser (https://soma.weill.cornell.edu/apps/I4_Multiome/), and the microbiome browser (https://soma.weill.cornell.edu/apps/I4_Microbiome/). For the PBMC data, the data are available with OSDR accession ID: OSD-570 and the following link: https://osdr.nasa.gov/bio/repo/data/studies/OSD-570/. Lastly, the data utilized for the specific sarcopenia analysis was accessed from archived data on the Gene Expression Omnibus (GEO) with accension ID GSE111017 and the following link: https://www.ncbi.nlm.nih.gov/geo/query/acc.cgi?acc=GSE111017.

## Abbreviations

BP: Biological processes
CFE: Cell-free epigenome
DEGs: Differentially expressed genes
EDL: Extensor digitorum longus
FBA: Flux balance analysis
GO: Gene ontology
GSEA: Gene set enrichment analysis
I4: Inspiration4
ISS: International Space Station
JAXA: Japan Aerospace Exploration Agency
L-: Days before spacecraft Lunch
MAS: Mean accuracy score
MF: Molecular function
MSigDB: Molecular signatures database
NASA: National Aeronautics and Space Administration
NES: Normalized enrichment score
OSD: Open science data
OSDR: NASA’s open science data repository
padj: Adjusted *p*-value
PBMCs: Peripheral blood mononuclear cells
R+: Days after spacecraft Re-entry RNA-seq, RNA-sequencing
