# Supplementary material for: Aging and putative frailty biomarkers are altered by spaceflight
Source: Sci Rep. 2024 Jun 11;14:13098. doi: 10.1038/s41598-024-57948-5 (PMC11166946; doi:10.1038/s41598-024-57948-5)
Supplement: Supplementary file 1 — Dataset S1. [file 41598_2024_57948_MOESM1_ESM.docx]

| **Mouse gene name** | **Mouse gene stable ID** | **Human Gene name** | **Gene stable ID** |
| --- | --- | --- | --- |
| [Cx3cl1](http://www.ensembl.org/mus_musculus/Gene/Summary?g=ENSMUSG00000031778) | [ENSMUSG00000031778](http://www.ensembl.org/mus_musculus/Gene/Summary?g=ENSMUSG00000031778) | [CX3CL1](http://www.ensembl.org/homo_sapiens/Gene/Summary?db=core;g=ENSG00000006210) | [ENSG00000006210](http://www.ensembl.org/homo_sapiens/Gene/Summary?db=core;g=ENSG00000006210) |
| [Nos2](http://www.ensembl.org/mus_musculus/Gene/Summary?g=ENSMUSG00000020826) | [ENSMUSG00000020826](http://www.ensembl.org/mus_musculus/Gene/Summary?g=ENSMUSG00000020826) | [NOS2](http://www.ensembl.org/homo_sapiens/Gene/Summary?db=core;g=ENSG00000007171) | [ENSG00000007171](http://www.ensembl.org/homo_sapiens/Gene/Summary?db=core;g=ENSG00000007171) |
| [Vim](http://www.ensembl.org/mus_musculus/Gene/Summary?g=ENSMUSG00000026728) | [ENSMUSG00000026728](http://www.ensembl.org/mus_musculus/Gene/Summary?g=ENSMUSG00000026728) | [VIM](http://www.ensembl.org/homo_sapiens/Gene/Summary?db=core;g=ENSG00000026025) | [ENSG00000026025](http://www.ensembl.org/homo_sapiens/Gene/Summary?db=core;g=ENSG00000026025) |
| [Fas](http://www.ensembl.org/mus_musculus/Gene/Summary?g=ENSMUSG00000024778) | [ENSMUSG00000024778](http://www.ensembl.org/mus_musculus/Gene/Summary?g=ENSMUSG00000024778) | [FAS](http://www.ensembl.org/homo_sapiens/Gene/Summary?db=core;g=ENSG00000026103) | [ENSG00000026103](http://www.ensembl.org/homo_sapiens/Gene/Summary?db=core;g=ENSG00000026103) |
| [Vcan](http://www.ensembl.org/mus_musculus/Gene/Summary?g=ENSMUSG00000021614) | [ENSMUSG00000021614](http://www.ensembl.org/mus_musculus/Gene/Summary?g=ENSMUSG00000021614) | [VCAN](http://www.ensembl.org/homo_sapiens/Gene/Summary?db=core;g=ENSG00000038427) | [ENSG00000038427](http://www.ensembl.org/homo_sapiens/Gene/Summary?db=core;g=ENSG00000038427) |
| [Ptgs2](http://www.ensembl.org/mus_musculus/Gene/Summary?g=ENSMUSG00000032487) | [ENSMUSG00000032487](http://www.ensembl.org/mus_musculus/Gene/Summary?g=ENSMUSG00000032487) | [PTGS2](http://www.ensembl.org/homo_sapiens/Gene/Summary?db=core;g=ENSG00000073756) | [ENSG00000073756](http://www.ensembl.org/homo_sapiens/Gene/Summary?db=core;g=ENSG00000073756) |
| [Arg2](http://www.ensembl.org/mus_musculus/Gene/Summary?g=ENSMUSG00000021125) | [ENSMUSG00000021125](http://www.ensembl.org/mus_musculus/Gene/Summary?g=ENSMUSG00000021125) | [ARG2](http://www.ensembl.org/homo_sapiens/Gene/Summary?db=core;g=ENSG00000081181) | [ENSG00000081181](http://www.ensembl.org/homo_sapiens/Gene/Summary?db=core;g=ENSG00000081181) |
| [Sirt1](http://www.ensembl.org/mus_musculus/Gene/Summary?g=ENSMUSG00000020063) | [ENSMUSG00000020063](http://www.ensembl.org/mus_musculus/Gene/Summary?g=ENSMUSG00000020063) | [SIRT1](http://www.ensembl.org/homo_sapiens/Gene/Summary?db=core;g=ENSG00000096717) | [ENSG00000096717](http://www.ensembl.org/homo_sapiens/Gene/Summary?db=core;g=ENSG00000096717) |
| [Hif1a](http://www.ensembl.org/mus_musculus/Gene/Summary?g=ENSMUSG00000021109) | [ENSMUSG00000021109](http://www.ensembl.org/mus_musculus/Gene/Summary?g=ENSMUSG00000021109) | [HIF1A](http://www.ensembl.org/homo_sapiens/Gene/Summary?db=core;g=ENSG00000100644) | [ENSG00000100644](http://www.ensembl.org/homo_sapiens/Gene/Summary?db=core;g=ENSG00000100644) |
| [Jag1](http://www.ensembl.org/mus_musculus/Gene/Summary?g=ENSMUSG00000027276) | [ENSMUSG00000027276](http://www.ensembl.org/mus_musculus/Gene/Summary?g=ENSMUSG00000027276) | [JAG1](http://www.ensembl.org/homo_sapiens/Gene/Summary?db=core;g=ENSG00000101384) | [ENSG00000101384](http://www.ensembl.org/homo_sapiens/Gene/Summary?db=core;g=ENSG00000101384) |
| [Ahcyl](http://www.ensembl.org/mus_musculus/Gene/Summary?g=ENSMUSG00000048087) | [ENSMUSG00000048087](http://www.ensembl.org/mus_musculus/Gene/Summary?g=ENSMUSG00000048087) | [AHCY](http://www.ensembl.org/homo_sapiens/Gene/Summary?db=core;g=ENSG00000101444) | [ENSG00000101444](http://www.ensembl.org/homo_sapiens/Gene/Summary?db=core;g=ENSG00000101444) |
| [Ahcy](http://www.ensembl.org/mus_musculus/Gene/Summary?g=ENSMUSG00000027597) | [ENSMUSG00000027597](http://www.ensembl.org/mus_musculus/Gene/Summary?g=ENSMUSG00000027597) | [AHCY](http://www.ensembl.org/homo_sapiens/Gene/Summary?db=core;g=ENSG00000101444) | [ENSG00000101444](http://www.ensembl.org/homo_sapiens/Gene/Summary?db=core;g=ENSG00000101444) |
| [Hmox2](http://www.ensembl.org/mus_musculus/Gene/Summary?g=ENSMUSG00000004070) | [ENSMUSG00000004070](http://www.ensembl.org/mus_musculus/Gene/Summary?g=ENSMUSG00000004070) | [HMOX2](http://www.ensembl.org/homo_sapiens/Gene/Summary?db=core;g=ENSG00000103415) | [ENSG00000103415](http://www.ensembl.org/homo_sapiens/Gene/Summary?db=core;g=ENSG00000103415) |
| [Il7](http://www.ensembl.org/mus_musculus/Gene/Summary?g=ENSMUSG00000040329) | [ENSMUSG00000040329](http://www.ensembl.org/mus_musculus/Gene/Summary?g=ENSMUSG00000040329) | [IL7](http://www.ensembl.org/homo_sapiens/Gene/Summary?db=core;g=ENSG00000104432) | [ENSG00000104432](http://www.ensembl.org/homo_sapiens/Gene/Summary?db=core;g=ENSG00000104432) |
| [Oaz1](http://www.ensembl.org/mus_musculus/Gene/Summary?g=ENSMUSG00000035242) | [ENSMUSG00000035242](http://www.ensembl.org/mus_musculus/Gene/Summary?g=ENSMUSG00000035242) | [OAZ1](http://www.ensembl.org/homo_sapiens/Gene/Summary?db=core;g=ENSG00000104904) | [ENSG00000104904](http://www.ensembl.org/homo_sapiens/Gene/Summary?db=core;g=ENSG00000104904) |
| [Tgfb1](http://www.ensembl.org/mus_musculus/Gene/Summary?g=ENSMUSG00000002603) | [ENSMUSG00000002603](http://www.ensembl.org/mus_musculus/Gene/Summary?g=ENSMUSG00000002603) | [TGFB1](http://www.ensembl.org/homo_sapiens/Gene/Summary?db=core;g=ENSG00000105329) | [ENSG00000105329](http://www.ensembl.org/homo_sapiens/Gene/Summary?db=core;g=ENSG00000105329) |
| [Fgf21](http://www.ensembl.org/mus_musculus/Gene/Summary?g=ENSMUSG00000030827) | [ENSMUSG00000030827](http://www.ensembl.org/mus_musculus/Gene/Summary?g=ENSMUSG00000030827) | [FGF21](http://www.ensembl.org/homo_sapiens/Gene/Summary?db=core;g=ENSG00000105550) | [ENSG00000105550](http://www.ensembl.org/homo_sapiens/Gene/Summary?db=core;g=ENSG00000105550) |
| [Lmbr1](http://www.ensembl.org/mus_musculus/Gene/Summary?g=ENSMUSG00000010721) | [ENSMUSG00000010721](http://www.ensembl.org/mus_musculus/Gene/Summary?g=ENSMUSG00000010721) | [LMBR1](http://www.ensembl.org/homo_sapiens/Gene/Summary?db=core;g=ENSG00000105983) | [ENSG00000105983](http://www.ensembl.org/homo_sapiens/Gene/Summary?db=core;g=ENSG00000105983) |
| [Tmem245](http://www.ensembl.org/mus_musculus/Gene/Summary?g=ENSMUSG00000055296) | [ENSMUSG00000055296](http://www.ensembl.org/mus_musculus/Gene/Summary?g=ENSMUSG00000055296) | [TMEM245](http://www.ensembl.org/homo_sapiens/Gene/Summary?db=core;g=ENSG00000106771) | [ENSG00000106771](http://www.ensembl.org/homo_sapiens/Gene/Summary?db=core;g=ENSG00000106771) |
| [Cxcl12](http://www.ensembl.org/mus_musculus/Gene/Summary?g=ENSMUSG00000061353) | [ENSMUSG00000061353](http://www.ensembl.org/mus_musculus/Gene/Summary?g=ENSMUSG00000061353) | [CXCL12](http://www.ensembl.org/homo_sapiens/Gene/Summary?db=core;g=ENSG00000107562) | [ENSG00000107562](http://www.ensembl.org/homo_sapiens/Gene/Summary?db=core;g=ENSG00000107562) |
| [Ppargc1a](http://www.ensembl.org/mus_musculus/Gene/Summary?g=ENSMUSG00000029167) | [ENSMUSG00000029167](http://www.ensembl.org/mus_musculus/Gene/Summary?g=ENSMUSG00000029167) | [PPARGC1A](http://www.ensembl.org/homo_sapiens/Gene/Summary?db=core;g=ENSG00000109819) | [ENSG00000109819](http://www.ensembl.org/homo_sapiens/Gene/Summary?db=core;g=ENSG00000109819) |
| [Cyp27b1](http://www.ensembl.org/mus_musculus/Gene/Summary?g=ENSMUSG00000006724) | [ENSMUSG00000006724](http://www.ensembl.org/mus_musculus/Gene/Summary?g=ENSMUSG00000006724) | [CYP27B1](http://www.ensembl.org/homo_sapiens/Gene/Summary?db=core;g=ENSG00000111012) | [ENSG00000111012](http://www.ensembl.org/homo_sapiens/Gene/Summary?db=core;g=ENSG00000111012) |
| [Krt18](http://www.ensembl.org/mus_musculus/Gene/Summary?g=ENSMUSG00000023043) | [ENSMUSG00000023043](http://www.ensembl.org/mus_musculus/Gene/Summary?g=ENSMUSG00000023043) | [KRT18](http://www.ensembl.org/homo_sapiens/Gene/Summary?db=core;g=ENSG00000111057) | [ENSG00000111057](http://www.ensembl.org/homo_sapiens/Gene/Summary?db=core;g=ENSG00000111057) |
| [Vdr](http://www.ensembl.org/mus_musculus/Gene/Summary?g=ENSMUSG00000022479) | [ENSMUSG00000022479](http://www.ensembl.org/mus_musculus/Gene/Summary?g=ENSMUSG00000022479) | [VDR](http://www.ensembl.org/homo_sapiens/Gene/Summary?db=core;g=ENSG00000111424) | [ENSG00000111424](http://www.ensembl.org/homo_sapiens/Gene/Summary?db=core;g=ENSG00000111424) |
| [Ifng](http://www.ensembl.org/mus_musculus/Gene/Summary?g=ENSMUSG00000055170) | [ENSMUSG00000055170](http://www.ensembl.org/mus_musculus/Gene/Summary?g=ENSMUSG00000055170) | [IFNG](http://www.ensembl.org/homo_sapiens/Gene/Summary?db=core;g=ENSG00000111537) | [ENSG00000111537](http://www.ensembl.org/homo_sapiens/Gene/Summary?db=core;g=ENSG00000111537) |
| [Ppard](http://www.ensembl.org/mus_musculus/Gene/Summary?g=ENSMUSG00000002250) | [ENSMUSG00000002250](http://www.ensembl.org/mus_musculus/Gene/Summary?g=ENSMUSG00000002250) | [PPARD](http://www.ensembl.org/homo_sapiens/Gene/Summary?db=core;g=ENSG00000112033) | [ENSG00000112033](http://www.ensembl.org/homo_sapiens/Gene/Summary?db=core;g=ENSG00000112033) |
| [Il17a](http://www.ensembl.org/mus_musculus/Gene/Summary?g=ENSMUSG00000025929) | [ENSMUSG00000025929](http://www.ensembl.org/mus_musculus/Gene/Summary?g=ENSMUSG00000025929) | [IL17A](http://www.ensembl.org/homo_sapiens/Gene/Summary?db=core;g=ENSG00000112115) | [ENSG00000112115](http://www.ensembl.org/homo_sapiens/Gene/Summary?db=core;g=ENSG00000112115) |
| [Il4](http://www.ensembl.org/mus_musculus/Gene/Summary?g=ENSMUSG00000000869) | [ENSMUSG00000000869](http://www.ensembl.org/mus_musculus/Gene/Summary?g=ENSMUSG00000000869) | [IL4](http://www.ensembl.org/homo_sapiens/Gene/Summary?db=core;g=ENSG00000113520) | [ENSG00000113520](http://www.ensembl.org/homo_sapiens/Gene/Summary?db=core;g=ENSG00000113520) |
| [Epas1](http://www.ensembl.org/mus_musculus/Gene/Summary?g=ENSMUSG00000024140) | [ENSMUSG00000024140](http://www.ensembl.org/mus_musculus/Gene/Summary?g=ENSMUSG00000024140) | [EPAS1](http://www.ensembl.org/homo_sapiens/Gene/Summary?db=core;g=ENSG00000116016) | [ENSG00000116016](http://www.ensembl.org/homo_sapiens/Gene/Summary?db=core;g=ENSG00000116016) |
| [Nfe2l2](http://www.ensembl.org/mus_musculus/Gene/Summary?g=ENSMUSG00000015839) | [ENSMUSG00000015839](http://www.ensembl.org/mus_musculus/Gene/Summary?g=ENSMUSG00000015839) | [NFE2L2](http://www.ensembl.org/homo_sapiens/Gene/Summary?db=core;g=ENSG00000116044) | [ENSG00000116044](http://www.ensembl.org/homo_sapiens/Gene/Summary?db=core;g=ENSG00000116044) |
| [Fasl](http://www.ensembl.org/mus_musculus/Gene/Summary?g=ENSMUSG00000000817) | [ENSMUSG00000000817](http://www.ensembl.org/mus_musculus/Gene/Summary?g=ENSMUSG00000000817) | [FASLG](http://www.ensembl.org/homo_sapiens/Gene/Summary?db=core;g=ENSG00000117560) | [ENSG00000117560](http://www.ensembl.org/homo_sapiens/Gene/Summary?db=core;g=ENSG00000117560) |
| [Fgf23](http://www.ensembl.org/mus_musculus/Gene/Summary?g=ENSMUSG00000000182) | [ENSMUSG00000000182](http://www.ensembl.org/mus_musculus/Gene/Summary?g=ENSMUSG00000000182) | [FGF23](http://www.ensembl.org/homo_sapiens/Gene/Summary?db=core;g=ENSG00000118972) | [ENSG00000118972](http://www.ensembl.org/homo_sapiens/Gene/Summary?db=core;g=ENSG00000118972) |
| [Plau](http://www.ensembl.org/mus_musculus/Gene/Summary?g=ENSMUSG00000021822) | [ENSMUSG00000021822](http://www.ensembl.org/mus_musculus/Gene/Summary?g=ENSMUSG00000021822) | [PLAU](http://www.ensembl.org/homo_sapiens/Gene/Summary?db=core;g=ENSG00000122861) | [ENSG00000122861](http://www.ensembl.org/homo_sapiens/Gene/Summary?db=core;g=ENSG00000122861) |
| [Hif3a](http://www.ensembl.org/mus_musculus/Gene/Summary?g=ENSMUSG00000004328) | [ENSMUSG00000004328](http://www.ensembl.org/mus_musculus/Gene/Summary?g=ENSMUSG00000004328) | [HIF3A](http://www.ensembl.org/homo_sapiens/Gene/Summary?db=core;g=ENSG00000124440) | [ENSG00000124440](http://www.ensembl.org/homo_sapiens/Gene/Summary?db=core;g=ENSG00000124440) |
| [Kdr](http://www.ensembl.org/mus_musculus/Gene/Summary?g=ENSMUSG00000062960) | [ENSMUSG00000062960](http://www.ensembl.org/mus_musculus/Gene/Summary?g=ENSMUSG00000062960) | [KDR](http://www.ensembl.org/homo_sapiens/Gene/Summary?db=core;g=ENSG00000128052) | [ENSG00000128052](http://www.ensembl.org/homo_sapiens/Gene/Summary?db=core;g=ENSG00000128052) |
| [Calu](http://www.ensembl.org/mus_musculus/Gene/Summary?g=ENSMUSG00000029767) | [ENSMUSG00000029767](http://www.ensembl.org/mus_musculus/Gene/Summary?g=ENSMUSG00000029767) | [CALU](http://www.ensembl.org/homo_sapiens/Gene/Summary?db=core;g=ENSG00000128595) | [ENSG00000128595](http://www.ensembl.org/homo_sapiens/Gene/Summary?db=core;g=ENSG00000128595) |
| [Egln3](http://www.ensembl.org/mus_musculus/Gene/Summary?g=ENSMUSG00000035105) | [ENSMUSG00000035105](http://www.ensembl.org/mus_musculus/Gene/Summary?g=ENSMUSG00000035105) | [EGLN3](http://www.ensembl.org/homo_sapiens/Gene/Summary?db=core;g=ENSG00000129521) | [ENSG00000129521](http://www.ensembl.org/homo_sapiens/Gene/Summary?db=core;g=ENSG00000129521) |
| [Ace2](http://www.ensembl.org/mus_musculus/Gene/Summary?g=ENSMUSG00000015405) | [ENSMUSG00000015405](http://www.ensembl.org/mus_musculus/Gene/Summary?g=ENSMUSG00000015405) | [ACE2](http://www.ensembl.org/homo_sapiens/Gene/Summary?db=core;g=ENSG00000130234) | [ENSG00000130234](http://www.ensembl.org/homo_sapiens/Gene/Summary?db=core;g=ENSG00000130234) |
| [Mas1](http://www.ensembl.org/mus_musculus/Gene/Summary?g=ENSMUSG00000068037) | [ENSMUSG00000068037](http://www.ensembl.org/mus_musculus/Gene/Summary?g=ENSMUSG00000068037) | [MAS1](http://www.ensembl.org/homo_sapiens/Gene/Summary?db=core;g=ENSG00000130368) | [ENSG00000130368](http://www.ensembl.org/homo_sapiens/Gene/Summary?db=core;g=ENSG00000130368) |
| [Gdf15](http://www.ensembl.org/mus_musculus/Gene/Summary?g=ENSMUSG00000038508) | [ENSMUSG00000038508](http://www.ensembl.org/mus_musculus/Gene/Summary?g=ENSMUSG00000038508) | [GDF15](http://www.ensembl.org/homo_sapiens/Gene/Summary?db=core;g=ENSG00000130513) | [ENSG00000130513](http://www.ensembl.org/homo_sapiens/Gene/Summary?db=core;g=ENSG00000130513) |
| [Sesn2](http://www.ensembl.org/mus_musculus/Gene/Summary?g=ENSMUSG00000028893) | [ENSMUSG00000028893](http://www.ensembl.org/mus_musculus/Gene/Summary?g=ENSMUSG00000028893) | [SESN2](http://www.ensembl.org/homo_sapiens/Gene/Summary?db=core;g=ENSG00000130766) | [ENSG00000130766](http://www.ensembl.org/homo_sapiens/Gene/Summary?db=core;g=ENSG00000130766) |
| [Rgn](http://www.ensembl.org/mus_musculus/Gene/Summary?g=ENSMUSG00000023070) | [ENSMUSG00000023070](http://www.ensembl.org/mus_musculus/Gene/Summary?g=ENSMUSG00000023070) | [RGN](http://www.ensembl.org/homo_sapiens/Gene/Summary?db=core;g=ENSG00000130988) | [ENSG00000130988](http://www.ensembl.org/homo_sapiens/Gene/Summary?db=core;g=ENSG00000130988) |
| [Lgals3](http://www.ensembl.org/mus_musculus/Gene/Summary?g=ENSMUSG00000050335) | [ENSMUSG00000050335](http://www.ensembl.org/mus_musculus/Gene/Summary?g=ENSMUSG00000050335) | [LGALS3](http://www.ensembl.org/homo_sapiens/Gene/Summary?db=core;g=ENSG00000131981) | [ENSG00000131981](http://www.ensembl.org/homo_sapiens/Gene/Summary?db=core;g=ENSG00000131981) |
| **Mouse gene name** | **Mouse gene stable ID** | **Human Gene name** | **Gene stable ID** |
| [Crp](http://www.ensembl.org/mus_musculus/Gene/Summary?g=ENSMUSG00000037942) | [ENSMUSG00000037942](http://www.ensembl.org/mus_musculus/Gene/Summary?g=ENSMUSG00000037942) | [CRP](http://www.ensembl.org/homo_sapiens/Gene/Summary?db=core;g=ENSG00000132693) | [ENSG00000132693](http://www.ensembl.org/homo_sapiens/Gene/Summary?db=core;g=ENSG00000132693) |
| [Kl](http://www.ensembl.org/mus_musculus/Gene/Summary?g=ENSMUSG00000058488) | [ENSMUSG00000058488](http://www.ensembl.org/mus_musculus/Gene/Summary?g=ENSMUSG00000058488) | [KL](http://www.ensembl.org/homo_sapiens/Gene/Summary?db=core;g=ENSG00000133116) | [ENSG00000133116](http://www.ensembl.org/homo_sapiens/Gene/Summary?db=core;g=ENSG00000133116) |
| [Snx14](http://www.ensembl.org/mus_musculus/Gene/Summary?g=ENSMUSG00000032422) | [ENSMUSG00000032422](http://www.ensembl.org/mus_musculus/Gene/Summary?g=ENSMUSG00000032422) | [SNX14](http://www.ensembl.org/homo_sapiens/Gene/Summary?db=core;g=ENSG00000135317) | [ENSG00000135317](http://www.ensembl.org/homo_sapiens/Gene/Summary?db=core;g=ENSG00000135317) |
| [Agt](http://www.ensembl.org/mus_musculus/Gene/Summary?g=ENSMUSG00000031980) | [ENSMUSG00000031980](http://www.ensembl.org/mus_musculus/Gene/Summary?g=ENSMUSG00000031980) | [AGT](http://www.ensembl.org/homo_sapiens/Gene/Summary?db=core;g=ENSG00000135744) | [ENSG00000135744](http://www.ensembl.org/homo_sapiens/Gene/Summary?db=core;g=ENSG00000135744) |
| [Rev1](http://www.ensembl.org/mus_musculus/Gene/Summary?g=ENSMUSG00000026082) | [ENSMUSG00000026082](http://www.ensembl.org/mus_musculus/Gene/Summary?g=ENSMUSG00000026082) | [REV1](http://www.ensembl.org/homo_sapiens/Gene/Summary?db=core;g=ENSG00000135945) | [ENSG00000135945](http://www.ensembl.org/homo_sapiens/Gene/Summary?db=core;g=ENSG00000135945) |
| [Il6](http://www.ensembl.org/mus_musculus/Gene/Summary?g=ENSMUSG00000025746) | [ENSMUSG00000025746](http://www.ensembl.org/mus_musculus/Gene/Summary?g=ENSMUSG00000025746) | [IL6](http://www.ensembl.org/homo_sapiens/Gene/Summary?db=core;g=ENSG00000136244) | [ENSG00000136244](http://www.ensembl.org/homo_sapiens/Gene/Summary?db=core;g=ENSG00000136244) |
| [Il10](http://www.ensembl.org/mus_musculus/Gene/Summary?g=ENSMUSG00000016529) | [ENSMUSG00000016529](http://www.ensembl.org/mus_musculus/Gene/Summary?g=ENSMUSG00000016529) | [IL10](http://www.ensembl.org/homo_sapiens/Gene/Summary?db=core;g=ENSG00000136634) | [ENSG00000136634](http://www.ensembl.org/homo_sapiens/Gene/Summary?db=core;g=ENSG00000136634) |
| [Capn11](http://www.ensembl.org/mus_musculus/Gene/Summary?g=ENSMUSG00000058626) | [ENSMUSG00000058626](http://www.ensembl.org/mus_musculus/Gene/Summary?g=ENSMUSG00000058626) | [CAPN11](http://www.ensembl.org/homo_sapiens/Gene/Summary?db=core;g=ENSG00000137225) | [ENSG00000137225](http://www.ensembl.org/homo_sapiens/Gene/Summary?db=core;g=ENSG00000137225) |
| [Mstn](http://www.ensembl.org/mus_musculus/Gene/Summary?g=ENSMUSG00000026100) | [ENSMUSG00000026100](http://www.ensembl.org/mus_musculus/Gene/Summary?g=ENSMUSG00000026100) | [MSTN](http://www.ensembl.org/homo_sapiens/Gene/Summary?db=core;g=ENSG00000138379) | [ENSG00000138379](http://www.ensembl.org/homo_sapiens/Gene/Summary?db=core;g=ENSG00000138379) |
| [Sh3gl3](http://www.ensembl.org/mus_musculus/Gene/Summary?g=ENSMUSG00000030638) | [ENSMUSG00000030638](http://www.ensembl.org/mus_musculus/Gene/Summary?g=ENSMUSG00000030638) | [SH3GL3](http://www.ensembl.org/homo_sapiens/Gene/Summary?db=core;g=ENSG00000140600) | [ENSG00000140600](http://www.ensembl.org/homo_sapiens/Gene/Summary?db=core;g=ENSG00000140600) |
| [Trp53](http://www.ensembl.org/mus_musculus/Gene/Summary?g=ENSMUSG00000059552) | [ENSMUSG00000059552](http://www.ensembl.org/mus_musculus/Gene/Summary?g=ENSMUSG00000059552) | [TP53](http://www.ensembl.org/homo_sapiens/Gene/Summary?db=core;g=ENSG00000141510) | [ENSG00000141510](http://www.ensembl.org/homo_sapiens/Gene/Summary?db=core;g=ENSG00000141510) |
| [Pmaip1](http://www.ensembl.org/mus_musculus/Gene/Summary?g=ENSMUSG00000024521) | [ENSMUSG00000024521](http://www.ensembl.org/mus_musculus/Gene/Summary?g=ENSMUSG00000024521) | [PMAIP1](http://www.ensembl.org/homo_sapiens/Gene/Summary?db=core;g=ENSG00000141682) | [ENSG00000141682](http://www.ensembl.org/homo_sapiens/Gene/Summary?db=core;g=ENSG00000141682) |
| [Akt1](http://www.ensembl.org/mus_musculus/Gene/Summary?g=ENSMUSG00000001729) | [ENSMUSG00000001729](http://www.ensembl.org/mus_musculus/Gene/Summary?g=ENSMUSG00000001729) | [AKT1](http://www.ensembl.org/homo_sapiens/Gene/Summary?db=core;g=ENSG00000142208) | [ENSG00000142208](http://www.ensembl.org/homo_sapiens/Gene/Summary?db=core;g=ENSG00000142208) |
| [Frem2](http://www.ensembl.org/mus_musculus/Gene/Summary?g=ENSMUSG00000037016) | [ENSMUSG00000037016](http://www.ensembl.org/mus_musculus/Gene/Summary?g=ENSMUSG00000037016) | [FREM2](http://www.ensembl.org/homo_sapiens/Gene/Summary?db=core;g=ENSG00000150893) | [ENSG00000150893](http://www.ensembl.org/homo_sapiens/Gene/Summary?db=core;g=ENSG00000150893) |
| [Mbip](http://www.ensembl.org/mus_musculus/Gene/Summary?g=ENSMUSG00000021028) | [ENSMUSG00000021028](http://www.ensembl.org/mus_musculus/Gene/Summary?g=ENSMUSG00000021028) | [MBIP](http://www.ensembl.org/homo_sapiens/Gene/Summary?db=core;g=ENSG00000151332) | [ENSG00000151332](http://www.ensembl.org/homo_sapiens/Gene/Summary?db=core;g=ENSG00000151332) |
| [Pglyrp3](http://www.ensembl.org/mus_musculus/Gene/Summary?g=ENSMUSG00000042244) | [ENSMUSG00000042244](http://www.ensembl.org/mus_musculus/Gene/Summary?g=ENSMUSG00000042244) | [PGLYRP3](http://www.ensembl.org/homo_sapiens/Gene/Summary?db=core;g=ENSG00000159527) | [ENSG00000159527](http://www.ensembl.org/homo_sapiens/Gene/Summary?db=core;g=ENSG00000159527) |
| [Ace](http://www.ensembl.org/mus_musculus/Gene/Summary?g=ENSMUSG00000020681) | [ENSMUSG00000020681](http://www.ensembl.org/mus_musculus/Gene/Summary?g=ENSMUSG00000020681) | [ACE](http://www.ensembl.org/homo_sapiens/Gene/Summary?db=core;g=ENSG00000159640) | [ENSG00000159640](http://www.ensembl.org/homo_sapiens/Gene/Summary?db=core;g=ENSG00000159640) |
| [Fndc5](http://www.ensembl.org/mus_musculus/Gene/Summary?g=ENSMUSG00000001334) | [ENSMUSG00000001334](http://www.ensembl.org/mus_musculus/Gene/Summary?g=ENSMUSG00000001334) | [FNDC5](http://www.ensembl.org/homo_sapiens/Gene/Summary?db=core;g=ENSG00000160097) | [ENSG00000160097](http://www.ensembl.org/homo_sapiens/Gene/Summary?db=core;g=ENSG00000160097) |
|  |  | [IVL](http://www.ensembl.org/homo_sapiens/Gene/Summary?db=core;g=ENSG00000163207) | [ENSG00000163207](http://www.ensembl.org/homo_sapiens/Gene/Summary?db=core;g=ENSG00000163207) |
| [Nos3](http://www.ensembl.org/mus_musculus/Gene/Summary?g=ENSMUSG00000028978) | [ENSMUSG00000028978](http://www.ensembl.org/mus_musculus/Gene/Summary?g=ENSMUSG00000028978) | [NOS3](http://www.ensembl.org/homo_sapiens/Gene/Summary?db=core;g=ENSG00000164867) | [ENSG00000164867](http://www.ensembl.org/homo_sapiens/Gene/Summary?db=core;g=ENSG00000164867) |
| [B2m](http://www.ensembl.org/mus_musculus/Gene/Summary?g=ENSMUSG00000060802) | [ENSMUSG00000060802](http://www.ensembl.org/mus_musculus/Gene/Summary?g=ENSMUSG00000060802) | [B2M](http://www.ensembl.org/homo_sapiens/Gene/Summary?db=core;g=ENSG00000166710) | [ENSG00000166710](http://www.ensembl.org/homo_sapiens/Gene/Summary?db=core;g=ENSG00000166710) |
| [Igfbp6](http://www.ensembl.org/mus_musculus/Gene/Summary?g=ENSMUSG00000023046) | [ENSMUSG00000023046](http://www.ensembl.org/mus_musculus/Gene/Summary?g=ENSMUSG00000023046) | [IGFBP6](http://www.ensembl.org/homo_sapiens/Gene/Summary?db=core;g=ENSG00000167779) | [ENSG00000167779](http://www.ensembl.org/homo_sapiens/Gene/Summary?db=core;g=ENSG00000167779) |
| [Cxcl10](http://www.ensembl.org/mus_musculus/Gene/Summary?g=ENSMUSG00000034855) | [ENSMUSG00000034855](http://www.ensembl.org/mus_musculus/Gene/Summary?g=ENSMUSG00000034855) | [CXCL10](http://www.ensembl.org/homo_sapiens/Gene/Summary?db=core;g=ENSG00000169245) | [ENSG00000169245](http://www.ensembl.org/homo_sapiens/Gene/Summary?db=core;g=ENSG00000169245) |
| [Bcl2l1](http://www.ensembl.org/mus_musculus/Gene/Summary?g=ENSMUSG00000007659) | [ENSMUSG00000007659](http://www.ensembl.org/mus_musculus/Gene/Summary?g=ENSMUSG00000007659) | [BCL2L1](http://www.ensembl.org/homo_sapiens/Gene/Summary?db=core;g=ENSG00000171552) | [ENSG00000171552](http://www.ensembl.org/homo_sapiens/Gene/Summary?db=core;g=ENSG00000171552) |
| [Ccl11](http://www.ensembl.org/mus_musculus/Gene/Summary?g=ENSMUSG00000020676) | [ENSMUSG00000020676](http://www.ensembl.org/mus_musculus/Gene/Summary?g=ENSMUSG00000020676) | [CCL11](http://www.ensembl.org/homo_sapiens/Gene/Summary?db=core;g=ENSG00000172156) | [ENSG00000172156](http://www.ensembl.org/homo_sapiens/Gene/Summary?db=core;g=ENSG00000172156) |
| [Lep](http://www.ensembl.org/mus_musculus/Gene/Summary?g=ENSMUSG00000059201) | [ENSMUSG00000059201](http://www.ensembl.org/mus_musculus/Gene/Summary?g=ENSMUSG00000059201) | [LEP](http://www.ensembl.org/homo_sapiens/Gene/Summary?db=core;g=ENSG00000174697) | [ENSG00000174697](http://www.ensembl.org/homo_sapiens/Gene/Summary?db=core;g=ENSG00000174697) |
| [Bdnf](http://www.ensembl.org/mus_musculus/Gene/Summary?g=ENSMUSG00000048482) | [ENSMUSG00000048482](http://www.ensembl.org/mus_musculus/Gene/Summary?g=ENSMUSG00000048482) | [BDNF](http://www.ensembl.org/homo_sapiens/Gene/Summary?db=core;g=ENSG00000176697) | [ENSG00000176697](http://www.ensembl.org/homo_sapiens/Gene/Summary?db=core;g=ENSG00000176697) |
| [Calr](http://www.ensembl.org/mus_musculus/Gene/Summary?g=ENSMUSG00000003814) | [ENSMUSG00000003814](http://www.ensembl.org/mus_musculus/Gene/Summary?g=ENSMUSG00000003814) | [CALR](http://www.ensembl.org/homo_sapiens/Gene/Summary?db=core;g=ENSG00000179218) | [ENSG00000179218](http://www.ensembl.org/homo_sapiens/Gene/Summary?db=core;g=ENSG00000179218) |
| [Rp1l1](http://www.ensembl.org/mus_musculus/Gene/Summary?g=ENSMUSG00000046049) | [ENSMUSG00000046049](http://www.ensembl.org/mus_musculus/Gene/Summary?g=ENSMUSG00000046049) | [RP1L1](http://www.ensembl.org/homo_sapiens/Gene/Summary?db=core;g=ENSG00000183638) | [ENSG00000183638](http://www.ensembl.org/homo_sapiens/Gene/Summary?db=core;g=ENSG00000183638) |
| [Gpr1](http://www.ensembl.org/mus_musculus/Gene/Summary?g=ENSMUSG00000046856) | [ENSMUSG00000046856](http://www.ensembl.org/mus_musculus/Gene/Summary?g=ENSMUSG00000046856) | [CMKLR2](http://www.ensembl.org/homo_sapiens/Gene/Summary?db=core;g=ENSG00000183671) | [ENSG00000183671](http://www.ensembl.org/homo_sapiens/Gene/Summary?db=core;g=ENSG00000183671) |
| [Tex38](http://www.ensembl.org/mus_musculus/Gene/Summary?g=ENSMUSG00000044556) | [ENSMUSG00000044556](http://www.ensembl.org/mus_musculus/Gene/Summary?g=ENSMUSG00000044556) | [TEX38](http://www.ensembl.org/homo_sapiens/Gene/Summary?db=core;g=ENSG00000186118) | [ENSG00000186118](http://www.ensembl.org/homo_sapiens/Gene/Summary?db=core;g=ENSG00000186118) |
| [Pax5](http://www.ensembl.org/mus_musculus/Gene/Summary?g=ENSMUSG00000014030) | [ENSMUSG00000014030](http://www.ensembl.org/mus_musculus/Gene/Summary?g=ENSMUSG00000014030) | [PAX5](http://www.ensembl.org/homo_sapiens/Gene/Summary?db=core;g=ENSG00000196092) | [ENSG00000196092](http://www.ensembl.org/homo_sapiens/Gene/Summary?db=core;g=ENSG00000196092) |
| [Adh4](http://www.ensembl.org/mus_musculus/Gene/Summary?g=ENSMUSG00000037797) | [ENSMUSG00000037797](http://www.ensembl.org/mus_musculus/Gene/Summary?g=ENSMUSG00000037797) | [ADH4](http://www.ensembl.org/homo_sapiens/Gene/Summary?db=core;g=ENSG00000198099) | [ENSG00000198099](http://www.ensembl.org/homo_sapiens/Gene/Summary?db=core;g=ENSG00000198099) |
| [Txnrd1](http://www.ensembl.org/mus_musculus/Gene/Summary?g=ENSMUSG00000020250) | [ENSMUSG00000020250](http://www.ensembl.org/mus_musculus/Gene/Summary?g=ENSMUSG00000020250) | [TXNRD1](http://www.ensembl.org/homo_sapiens/Gene/Summary?db=core;g=ENSG00000198431) | [ENSG00000198431](http://www.ensembl.org/homo_sapiens/Gene/Summary?db=core;g=ENSG00000198431) |
| [Mtor](http://www.ensembl.org/mus_musculus/Gene/Summary?g=ENSMUSG00000028991) | [ENSMUSG00000028991](http://www.ensembl.org/mus_musculus/Gene/Summary?g=ENSMUSG00000028991) | [MTOR](http://www.ensembl.org/homo_sapiens/Gene/Summary?db=core;g=ENSG00000198793) | [ENSG00000198793](http://www.ensembl.org/homo_sapiens/Gene/Summary?db=core;g=ENSG00000198793) |
| [Atxn2](http://www.ensembl.org/mus_musculus/Gene/Summary?g=ENSMUSG00000042605) | [ENSMUSG00000042605](http://www.ensembl.org/mus_musculus/Gene/Summary?g=ENSMUSG00000042605) | [ATXN2](http://www.ensembl.org/homo_sapiens/Gene/Summary?db=core;g=ENSG00000204842) | [ENSG00000204842](http://www.ensembl.org/homo_sapiens/Gene/Summary?db=core;g=ENSG00000204842) |
| [Rad51ap2](http://www.ensembl.org/mus_musculus/Gene/Summary?g=ENSMUSG00000086022) | [ENSMUSG00000086022](http://www.ensembl.org/mus_musculus/Gene/Summary?g=ENSMUSG00000086022) | [RAD51AP2](http://www.ensembl.org/homo_sapiens/Gene/Summary?db=core;g=ENSG00000214842) | [ENSG00000214842](http://www.ensembl.org/homo_sapiens/Gene/Summary?db=core;g=ENSG00000214842) |
| [Cntf](http://www.ensembl.org/mus_musculus/Gene/Summary?g=ENSMUSG00000079415) | [ENSMUSG00000079415](http://www.ensembl.org/mus_musculus/Gene/Summary?g=ENSMUSG00000079415) | [CNTF](http://www.ensembl.org/homo_sapiens/Gene/Summary?db=core;g=ENSG00000242689) | [ENSG00000242689](http://www.ensembl.org/homo_sapiens/Gene/Summary?db=core;g=ENSG00000242689) |
| [Actn3](http://www.ensembl.org/mus_musculus/Gene/Summary?g=ENSMUSG00000006457) | [ENSMUSG00000006457](http://www.ensembl.org/mus_musculus/Gene/Summary?g=ENSMUSG00000006457) | [ACTN3](http://www.ensembl.org/homo_sapiens/Gene/Summary?db=core;g=ENSG00000248746) | [ENSG00000248746](http://www.ensembl.org/homo_sapiens/Gene/Summary?db=core;g=ENSG00000248746) |
|  |  | [B2M](http://www.ensembl.org/homo_sapiens/Gene/Summary?db=core;g=ENSG00000273686) | [ENSG00000273686](http://www.ensembl.org/homo_sapiens/Gene/Summary?db=core;g=ENSG00000273686) |
|  |  | [HMOX2](http://www.ensembl.org/homo_sapiens/Gene/Summary?db=core;g=ENSG00000277424) | [ENSG00000277424](http://www.ensembl.org/homo_sapiens/Gene/Summary?db=core;g=ENSG00000277424) |
|  |  | [CMKLR2](http://www.ensembl.org/homo_sapiens/Gene/Summary?db=core;g=ENSG00000283448) | [ENSG00000283448](http://www.ensembl.org/homo_sapiens/Gene/Summary?db=core;g=ENSG00000283448) |
|  |  | [SESN2](http://www.ensembl.org/homo_sapiens/Gene/Summary?db=core;g=ENSG00000285069) | [ENSG00000285069](http://www.ensembl.org/homo_sapiens/Gene/Summary?db=core;g=ENSG00000285069) |
| **Mouse gene name** | **Mouse gene stable ID** | **Human Gene name** | **Gene stable ID** |
| [Sod2](http://www.ensembl.org/mus_musculus/Gene/Summary?g=ENSMUSG00000006818) | [ENSMUSG00000006818](http://www.ensembl.org/mus_musculus/Gene/Summary?g=ENSMUSG00000006818) | [SOD2](http://www.ensembl.org/homo_sapiens/Gene/Summary?db=core;g=ENSG00000291237) | [ENSG00000291237](http://www.ensembl.org/homo_sapiens/Gene/Summary?db=core;g=ENSG00000291237) |
