# Supplementary material for: Aging and putative frailty biomarkers are altered by spaceflight
Source: Sci Rep. 2024 Jun 11;14:13098. doi: 10.1038/s41598-024-57948-5 (PMC11166946; doi:10.1038/s41598-024-57948-5)
Supplement: Supplementary file 7 — Supplementary Legends. [file 41598_2024_57948_MOESM7_ESM.docx]

**Supplementary Material**

**Aging and putative frailty biomarkers are altered by spaceflight**

Andrea Camera, Marshall Tabetah, Veronica Castañeda, JangKeun Kim, Aman Singh Galsinh, Alissen Haro-Vinueza, Ivonne Salinas, Allen Seylani, Shehbeel Arif, Saswati Das, Marcelo A. Mori, Anthony Carano, Lorraine Christine De Oliveira, Masafumi Muratani, Richard Barker, Victoria Zaksas, Chirag Goel, Eleni Dimokidis, Deanne M. Taylor, Jisu Jeong, Eliah Overbey, Cem Meydan, D. Marshall Porterfield, Juan Esteban Díaz, Andrés Caicedo, Jonathan C. Schisler, Evagelia C. Laiakis, Christopher E. Mason, Man S Kim, Fathi Karouia, Nathaniel J Szewczyk, Afshin Beheshti

**Supplementary Data 1**. **List of frailty related genes.**

**Supplementary Data 2**. **The list of unique and comment genes for the mice and human data.**

**Supplementary Data 3**. **The GSEA pathway results displayed in Figure 3B.**

**Supplementary Data 4**. **The differential gene expression results for the sarcopenia study GSE111006, GSE111010, and GSE111016) utilized for Figure 4.**

**Supplementary Data 5**. **The GSEA pathway results displayed in Figure 3C.**

**Supplementary Data 6**. **The data for the metabolic flux analysis displayed in Figure 8**
